# Paraoxonase 2 overexpression inhibits tumor development in a mouse model of ovarian cancer

**DOI:** 10.1038/s41419-018-0395-2

**Published:** 2018-03-12

**Authors:** Asokan Devarajan, Feng Su, Victor Grijalva, Meghna Yalamanchi, Ashna Yalamanchi, Feng Gao, Hannah Trost, Josephine Nwokedi, Gina Farias-Eisner, Robin Farias-Eisner, Alan M. Fogelman, Srinivasa T Reddy

**Affiliations:** 1 Department of Medicine, David Geffen School of Medicine, University of California Los Angeles, Los Angeles, CA 90095-1736 USA; 2 Department of Obstetrics and Gynecology, David Geffen School of Medicine, University of California Los Angeles, Los Angeles, CA 90095-1736 USA; 3 Department of Surgery, David Geffen School of Medicine, University of California Los Angeles, Los Angeles, CA 90095-1736 USA; 4 Department of Molecular and Medical Pharmacology, David Geffen School of Medicine, University of California Los Angeles, Los Angeles, CA 90095-1736 USA; 5 Present Address: Clinical Medicine Research Center of Affiliated Hospital, Inner Mongolia Medical University, Hohhot, Inner Mongolia, China

## Abstract

Ovarian cancer (OC) is most lethal malignancy among all gynecological cancer. Large bodies of evidences suggest that mitochondrial-derived ROS play a critical role in the development and progression of OC. Paraoxonase 2 (PON2) is a membrane-associated lactonase with anti-oxidant properties. PON2 deficiency aggravates mitochondrial ROS formation, systemic inflammation, and atherosclerosis. The role of PON2 in cancer development remains unknown. In this report, in human, we identified that PON2 expression is higher in early stages (but not in late stages) of OC when compared to normal tissue. Using a mouse xenograft model of OC, we demonstrate that overexpression of PON2 prevents tumor formation. Mechanistically, PON2 decreases OC cell proliferation by inhibiting insulin like growth factor-1 (IGF-1) expression and signaling. Intriguingly, PON2 reduces c-Jun-mediated transcriptional activation of IGF-1 gene by decreasing mitochondrial superoxide generation. In addition, PON2 impairs insulin like growth factor-1 receptor (IGF-1R) signaling in OC cells by altering cholesterol homeostasis, which resulted in reduced caveolin-1/IGF-1R interaction and IGF-1R phosphorylation. Taken together, we report for the first time that PON2 acts as a tumor suppressor in the early stage of OC by reducing IGF-1 production and its signaling, indicating PON2 activation might be a fruitful strategy to inhibit early stage ovarian tumor.

## Introduction

The precise spatiotemporal control of reactive oxygen species (ROS) generation is a critical regulator of both cell survival and death. Mitochondrial oxidative stress and mitochondrial-derived ROS play an important role in the vitality of cancer cells and drive signal transduction pathways, which lead to activation of mitogenic growth factors, redox sensitive transcription factors, angiogenesis, and genes involved in cancer cell growth, proliferation, and survival^1–3^. Accumulating evidence, from both animal and human studies, suggests that mitochondrial-derived ROS^4^ play a critical role in the development and progression of OC^5^.

Paraoxonase 2 (PON2) belongs to the PON gene family, which consists of PON1, PON2, and PON3. All three PONs have anti-oxidant properties. PON1 is associated with HDL whereas, PON2 and PON3 are intracellular membrane proteins^6,7^. PON2 is detected in various organs and all types of cells including vascular cells^6,7^ and is localized in the inner mitochondrial membrane, where it associates with mitochondrial respiratory complex III, binds Coenzyme Q10, and regulates the respiratory complex activity and prevents the ubisemiquinone mediated mitochondrial superoxide levels and oxidative stress in vascular cells and the liver^8^. Knockout and transgenic mouse models have shown that PON2 protects against the development of atherosclerosis, obesity, insulin resistance, and neurogenerative diseases^6,9–13^.

PON2 has been shown to be upregulated in tumor tissues relative to corresponding normal tissues in many types of cancers^14^. However, the role and mechanism of action of PON2 in cancer has not been elucidated. In this report, we demonstrate that when injected into mice, ID8 cells (a mouse ovarian cancer cell line) overexpressing hPON2 (ID8^hPON2^) develop significantly reduced tumor size and volume compared to mice receiving empty vector-ID8 (ID8^EV^) cells. Utilizing molecular, biochemical, and immunological approaches, we demonstrate that PON2 decreases ovarian cancer cell proliferation by regulating both IGF-1 expression as well as IGF-1 signaling. We show that the reduction in IGF-1 levels is c-Jun-dependent and associated with decreased mitochondrial superoxide levels. Moreover, independent of IGF-1 levels, PON2 expression alters the IGF-1 signaling by reducing caveolin-1/IGF-1R interaction and IGF-1R phosphorylation. Our results suggest that PON2 overexpression reduces the tumor forming potential of ID8 cells by reducing the IGF-1 signaling and its signaling pathway.

## Material and methods

### Reagents and cell culture

ID8-cells were transfected with either a pcDNA 3.1 vector carrying a human PON2 cDNA (hPON2) or pcDNA 3.1 vector alone and stable cell lines (ID8^hPON2^ and ID8^EV^, respectively) were established^15^. ID8^hPON2^ and ID8^EV^ cells were routinely cultured in Dulbecco’s Modified Eagles Medium (DMEM) with high glucose and l-glutamine (2 mM), supplemented with 4% fetal bovine serum (FBS), penicillin (100 U ml^−1^), streptomycin (100 μg ml^−1^), 1× insulin, transferrin, sodium selenite (ITS) liquid media supplement (Sigma-Aldrich, St. Louis, MO), and G418 (800 μg ml^−1^). The individual experimental treatments for ID8^hPON2^ and ID8^EV^ cells were described in detail under the corresponding figure legends. OVCAR-5 cells were transiently transfected with either a pcDNA 3.1 vector carrying a human PON2 cDNA (hPON2) or pcDNA 3.1 vector alone to generate OVCAR-5^hPON2^ and OVCAR-5^EV^ cells, respectively that were cultured in RPMI 1640 medium, supplemented with 10% FBS and penicillin (100 U ml^−1^), streptomycin (100 μg ml^−1^). Two days post transfection, experiments were performed as described in the figure legends. We obtained ovarian cancer tissue (Stage I, Stage II, Stage III, and Stage IV) and normal adjacent tissue through the Gynecological oncology group and Cooperative human tissue network.

### Tissue array studies

The ovarian carcinoma tissue array (#BC11115a and #OV1004, US Biomax) slides were placed in xylenes to remove the paraffin, then a series of ethanol. Subsequently the slide was blocked with 3% H_2_O_2_/methanol for 10 min to inhibit the endogenous peroxidase activity. After a wash in distilled water, the slide was incubated for 25 min with EDTA buffer pH 8.00 at 95 °C using Decloaking Chamber NxGen (Biocare Medical, DC2012). Tissue section was incubated with rabbit PON2 antibody (#GTX104061, GeneTex) at the dilution of 1:100 at 4 °C for overnight. After washing with PBS containing 0.1% Tween 20, the section was stained with Dako EnVision System-HRP Labelled Polymer Anti-Rabbit (Dako, K4003) at room temperature for 30 min. Immunostaining was visualized using 3,3′-diaminobenzidine (DAB). Subsequently the slide was washed with tap water, counterstained with Harris’ Hematoxylin, dehydrated in ethanol, and mounted with media. Slide was digitized on a ScanScope AT (Leica Biosystems, Inc., Vista, CA) and morphometric analysis performed with Definiens’ Tissue Studio (Definiens Inc., Parsippany, NJ) to determine the percentage of PON2-positive cells in a non-biased method. Briefly, a stain-specific algorithm was created using the pre-defined cellular detection module and classification tool, positive and negative stained cells within a core were identified. Thresholds were set to classify hematoxylin stain for nuclei and DAB stain for positive cellular staining. The data were exported to Excel for further statistical analysis.

### Microarray studies

GeneChip Mouse Genome 430 2.0 Arrays (Affymetrix, Santa Clara, CA) were used to profile the transcriptomes in ID8^hPON2^ and ID8^EV^ cell lines. Briefly, biotinylated complementary RNA (cRNA) was prepared from 100 ng total RNA. Following fragmentation, 10 μg of cRNA was hybridized for 16 h at 45 °C on the microarray. GeneChips were washed and stained in the Affymetrix Fluidics Station 450 and then scanned using the GeneChip Scanner 3000 7 G. Quality control and a gene level robust multiarray average normalization were performed with the Affymetrix Expression Console software (version 1.3)

### Western blot analysis

Cell lysates were collected in lysis buffer containing 0.1 M NaCl, 5 mM EDTA, 50 μM sodium orthovanadate, 1% Triton X-100, and protease inhibitor tablet (Roche Diagnostics, Indianapolis, IN) in 50 mM Tris buffer, pH 7.5. Tissue was homogenized with lysis buffer, centrifuged, and supernatant was taken for experiments. Fifty micrograms of total protein per sample (except for ERK studies where we used 100 μg) were resolved on 4–15% SDS-PAGE gels, transferred onto nitrocellulose membranes for 1 h, and blocked in TBS containing 3% milk protein for 1 h. hPON2 was detected using goat hPON2 antibody (#AF4344, R&D Systems) at 1:500 dilutions. Rabbit IGF-1R beta (#9750), phospho-IGF-1R beta (#3918), and rabbit cyclin D1 (#2978) antibodies (Cell Signaling Technology) were also used at 1:500 dilution. Actin (#A2228) and Vinculin (#V9131) antibodies (Sigma Aldrich) were used at 1:5000 dilution. Primary antibodies were diluted and incubated in TBS containing 5% milk protein at 4 °C overnight. The membranes were washed, incubated with their respective secondary antibodies (1:5000) for 1 h, and proteins were illuminated using an ECL Plus Western blotting kit (GE Healthcare, UK).

### Subcellular localization studies

To examine whether hPON2 is localized in the mitochondria and ER of ID8^hPON2^ cells, cells were grown in cover glass with 12 well plates. To detect the mitochondria, cells were treated with Mito Tracker® Red (# M7512, Thermo Fisher Scientific) for 1 h. After fixation and permeabilization, cells were blocked with 1% goat serum albumin for 1 h at RT and then stained with mouse PON2 antibody (# ab85340, Abcam) for 1 h. Following three washes with phosphate buffer saline with 0.1% Triton, cells were incubated with anti-rabbit IgG FITC (1:50 dilution) for 1 h in the dark. To detect the hPON2 in ER, the experiment was performed as described before. Instead of Mito Tracker® Red, mouse calnexin antibody (# sc-23954,  Santa Cruz Biotechnology Inc.) and anti-mouse Rhodamine antibody were used for ER marker. Images were captured with a fluorescence microscope (Olympus IX70). Subcellular organelles were isolated from ID8^hPON2^ cells as described previously and hPON2 was detected with western blotting (#AF4344, R&D Systems) as described earlier.

### Mitochondrial respiratory complex II+III activity

In total, 100 μg mitochondrial protein from ID8^EV^ and ID8^hpon2^ was added with final a concentration of 20 μM succinate as substrates, 50 μM cytochrome c3+, and 250 μM of potassium cyanide, and the enzymatic activity was determined at 550 nm (*ɛ* = 18.5 mM^−1^ cm^−1^ ) and activity was expressed as nanomole cytochrome c reduced per minute per milligram protein.

### Citrate lyase activity

In total, 100 μg cytosolic protein from ID8^EV^ and ID8^hpon2^ was added with 100 mM triethanolamine buffer, 30 mM zinc chloride, 10 mM NADH, and 0.50 mM sodium citrate as substrate and enzyme activity was monitored at 340 nM and activity was expressed nanomole of oxaloacetate formed per minute per milligram protein.

### SiRNA silencing

HeLa, SKOV3, and A549 cultures at about 70% confluence were transfected with either 25 nM of control siRNA (AllStars Negative Control siRNA, Qiagen), or a pooled mixture (25 nM each) of the two PON2-specific siRNA (Hs_PON2_2 HP siRNA, Hs_PON2_6 HP, Qiagen). Transfection was performed using the Lipofectamine 2000 kit from Invitrogen according to the manufacturer’s recommended protocol. Cells were used in experiments 2 days after siRNA transfection.

### Cell proliferation assay using spectrophotometry

Cell proliferation was determined using pyrimidine analog, 5-bromo-2′-deoxyuridine (BrdU) as described in manufacturer’s protocol (Roche Applied Science, IN). Briefly, ID8^EV^ and ID8^hPON2^ cells were cultured in 96-well plates and treated as described under figure legends. BrdU (10 μM) was added to the cells for 2 h; cells were fixed and incubated with an anti-BrdU antibody-conjugated peroxidase for 2 h at 37 °C. Following antibody incubation, tetramethyl-benzidine substrate solution was added, the reaction was stopped at the appropriate time by adding 50 μl of 0.5 M sulfuric acid, and the absorbance value was detected at 450 nm using a plate reader (BMG Labtech, CA). All experiments were performed in triplicates; and data were expressed as the mean of the triplicate. Cell proliferation assays were also performed using a FITC BrdU flow kit (BD-Bioscience, San Jose, USA) according to manufacturer’s protocol.

### Cell proliferation assay and hPON2 detection using flow cytometer

Following hPON2 siRNA transfection with SKOV3, HeLa, and A549 cells, cell proliferation was assessed using FITC BrdU flow kit (#51-2354AK, BD Pharmingen). Briefly, BrdU pulsed cells (1 × 10^6^ cells) were resuspended in cytofix/cytoperm buffer at room temperature for 30 min. Following washes, cells were resuspended with cyto-permeabilization plus buffer for 10 min on ice and then refixed for 5 min. Following washing with buffer, the cells were treated with DNAase for 1 h at 37 °C. After adding fluorescent antibodies, Brdu-positive cells were quantified using flow cytometer. For hPON2 detection, cells were resuspended in cytofix/cytoperm buffer at room temperature for 30 min. Following washes, cells were resuspended with cyto-permeabilization plus buffer for 10 min on ice and then refixed for 5 min. Following washing with buffer, mouse PON2 antibody at 1:100 (#ab85340, Abcam) was incubated for 1 h subsequently FITC conjugated secondary antibody was incubated for 30 min and cells were quantified using flow cytometer.

### Cell viability assay

Cell viability assay was measured by MTS method (#ab197010, Abcam) as described in the manufacture protocol. ID8^EV^ and ID8h^PON2^ cells were seeded in 96-well plates at a density of 2 × 10^3^ per well and mixed with 20 μl of MTS dye, and then incubated for 30 min in the dark. The optical density (OD) values were measured at 490 nm using BMG labtech plate reader.

### Quantitative real-time PCR analysis (qRT-PCR)

ID8^EV^ and ID8^hPON2^ cells were cultured in six-well plates and treated as described under figure legends. Total RNA was extracted from ID8^hPON2^ and ID8^EV^ cells using Qiagen RNA isolation kit. The quality and quantity of RNA were analyzed on a NanoDrop system. cDNA was synthesized from a high capacity cDNA Reverse Transcription Kit according to the manufacturer’s protocol (Applied Biosystems, Foster City, CA). Two microliters of the cDNA was used for the PCR reaction with gene-specific primers and Q SYBR Green Supermix (BIO-RAD, Hercules, CA, USA) or green Supermix with Rox (VWR, Chicago, IL, USA) in a MyiQ Single-Color Real-Time PCR Detection System (BIO-RAD, Hercules, CA, USA). The cycling conditions were as follows: 3 min at 95 °C followed by 40 cycles of: 95 °C, 30 s; 60 °C, 1 min; 72 °C, 1 min; followed by a final extension at 72 °C for 10 min. The primer pairs were as follows: (1) mIGF1 (forward), GTCTTCACATCTCTTCTACCT and (reverse) AGCAACACTCATCCACAAT, (2) mCyclophilin (forward) GGCCGATGACGAGCCC and (reverse) TGTCTTTGGAACTTTGTCTGCAA, (3) hIGF-1 (forward) TGGATGCTCTTCAGTTCGTG and (reverse) TGGTAGATGGGGGCTGATAC, and (4) Beta actin (forward) GCATCCATGAAACTACATT and (reverse) CACTTGCGGTGCACGATGG.

### Intracellular ROS and mitochondrial ROS measurement

Total ROS was quantified using a 2′,7′-dichlorofluorescein (DCF) assay as previously described^16,17^. ID^EV^ and ID8^hPON2^ cells were plated and treated as described in the Materials and methods sections. Following serum starvation, cells were incubated with 100 µM DCF (Invitrogen, Carlsbad, CA) for 1 h at 37 °C. Cells were washed with Krebs-Ringer buffer. Fluorescence was measured by a fluorescence microplate reader (Spectra Max Gemini XS, Molecular Devices, Sunnyvale, CA) with an excitation filter at 485 nm and an emission filter at 530 nm. Mitochondrial superoxide levels were detected as described^8^.

### Immunoprecipitation

Briefly, 500 μg of protein lysate from ID8^EV^, ID8^hPON2^, scrambled siRNA-treated, and PON2 siRNA-treated cells were incubated with Caveolin-1 antibody (# 3267S,  Cell Signaling Technology, Danvers, MA) for overnight at 4 °C. Subsequently, antigen–antibody complexes were incubated with Protein A agarose beads for 3 h at 4 °C. Following three washes, pellets were resuspended with 3× SDS sample Buffer. Negative control was performed without antibody. IGF-1R was detected by western blotting. Caveolin-1 and IGF-1R were also detected from total lysate (50 μg protein) as input controls.

### Enzyme-linked immunosorbent assay

ID8^EV^ and ID8^PON2^ cells were grown 2–4 days and each day serum starved for 12 h. Media was collected and IGF-1 levels were measured following manufacturer’s protocol using mouse IGF-1 ELISA Kit (# ab100695, Abcam). Briefly, loading buffer was used to dilute serum and cell culture medium 1:4 and 1:2, respectively, followed by overnight incubation at 4 °C. After washing three times with washing buffer, 1× biotinylated IGF-1 antibody was added to each well and incubated for 1 h at RT with gentle shaking. Following this, wells were washed three times with washing buffer and 100 μL of 1× HRP-Streptavidin solution was added. TMB solution (100 μL) was added, and was incubated for 30 min at RT in the dark room. Color density was measured at 450 nm after adding stop solution. Plasma IGF-1 levels were quantified as described above.

### Chromatin immunoprecipitation assay

Chromatin immunoprecipitation (ChIP) assay was performed as described in the manufacturer’s instruction manual (Active Motif, Carlsbad, California). Briefly, ID8^EV^ and ID8^hPON2^ cells were grown in 15 cm plates. After 24 h (70–80% confluence), cells were serum starved for 12 h. Cells were fixed with fixing solution (0.54 ml of 37% formaldehyde in 20 ml medium) at RT for 20 min to cross-link the DNA with bound proteins. After washing, cells were resuspended in lysis buffer containing protease inhibitors and sonicated to shear the chromatin. The chromatin was further immunoprecipitated with C-Jun (#9165), p53 (#2524) (Cell signaling technology, Danvers, MA), and rabbit or mouse normal IgG as a control. Chromatin was harvested after immunoprecipitation, and then the cross-links were reversed and the DNA was purified and precipitated. The resulting DNA was measured primers (Qiagen, C-Jun binding site primer cat# GPM10289 (+)01A) and p53 binding site primer GPM1028976(−)01A)) and used as a template for the real-time PCR. The DNA enrichment after ChIP was estimated as the percentage bound-to-input ratio.

**Generation of flank tumors in C57BL/6J mice:** Six-week-old female C57BL/6J (Jackson laboratories) mice were maintained in a pathogen-free animal facility. All experiments were done in accordance with institutional guidelines. The mice were housed in a 12–12 h light–dark schedule and provided food and water ad libitum. ID8^hPON2^ or ID8^EV^ cells (5 × 10^6^/mice) were injected subcutaneously in the right flank of C57BL/6J (*n* = 15 per group). At the end of the 5th week, serum was collected, mice were sacrificed, and tumors were resected. Tumor size and volume were determined by Vernier calipers and using a formula (1/2 × *L* × *W*^2^) mm^3^.

### Acetyl CoA measurement

Cytosol and mitochondria were isolated as described previously^18^. Acetyl CoA was measured using a kit according to manufacturer’s protocol (#K317, Biovision). For normalization, protein content was measured in the samples prior to perchloric acid treatment.

### Cholesterol measurement

Lipids were extracted from cell lysates and conditioned medium collected from ID8^EV^, ID8^hPON2^, OVCAR5^EV^, OVCAR5^hPON2^, scrambled siRNA-treated, and PON2 siRNA-treated ID8 cells. Briefly, cells were homogenized with methanol, isopropanol, and Triton X100. Since phenol red is interfered with cholesterol reagent, lipids were extracted and then cholesterol was measured as described in the manufacturer’s protocol (# C7510, Pointe Scientific).

### Protein estimation

Protein was measured by Bradford method (Sigma Aldrich, St. Louis, USA).

### Statistical analysis

Data was expressed as mean ± SEM where indicated. Statistical differences were analyzed using the Student’s *t*-test or Anova and *p* values less than 0.05 were considered statistically significant.

## Results

### PON2 protein expression is upregulated in ovarian cancer

**Fig. 1:**
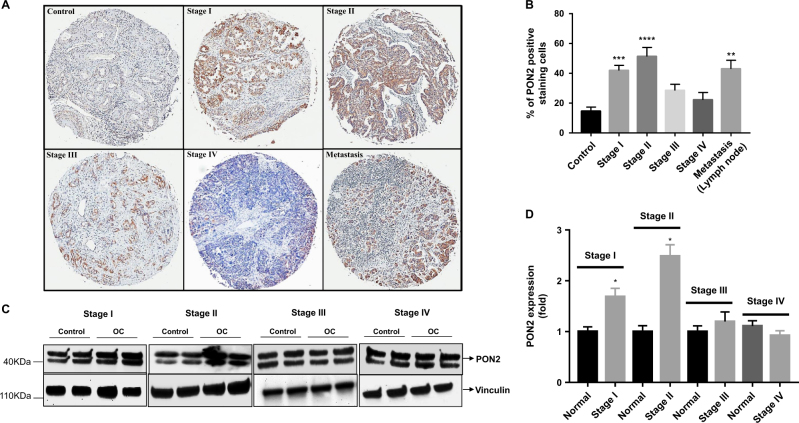
PON2 protein is induced in early stage ovarian cancer. Immunohistochemistry was performed on ovarian carcinoma tissue array slides (US Biomax Rockville, MD, USA) using PON2 antibody as described under the Materials and methods section. PON2 protein expression was quantified using Definiens Tissue Studio Software. Representative photographs of the staining are shown in **a**, and the quantification of the data is presented in **b**. Values are expressed as percentage of PON2-positive staining cells. Total protein lysates from matched normal and ovarian tumor tissues [stage I (*n* = 7), stage II (*n* = 10), stage III (*n* = 12), and stage IV (*n* = 15)] were subjected western blotting analyses using hPON2 antibody. Representative blots (two sets from each stage) are shown in **c**. The data were quantified using Image J software and presented in **d**

PON2 mRNA expression is upregulated in a number of tumor tissues compared to their corresponding normal tissues^19^. However, PON2 protein expression has not been examined at various stages of tumor development. In the present study, we analyzed PON2 protein expression by immunohistochemistry using ovarian cancer tissue arrays containing stage I (*n* = 23), stage II (*n *= 20), stage III (*n* = 35), stage IV (*n* = 12), metastasis (*n* = 10), and control (*n* = 20) tissues. Our results show that PON2 protein expression is significantly higher in stage I, stage II, and metastasis tissues (Fig. 1a, b). There was no significant change in PON2 expression in stage IV tissues when compared to normal tissue (Fig. 1a, b). Although PON2 expression was increased in stage III, it did not reach statistical significance when data from all 35 samples were averaged and compared to control group (*n* = 20) (Fig. 1b). Western blotting data showed that PON2 protein expression (observed as a doublet due to the two isoforms present in humans^20^) is significantly higher in stage I and stage II when compared to adjacent normal tissue, whereas there was no significant change in stage III and stage IV (Fig. 1c, d).

### Establishment of the stable ID8^EV^ and ID8^hPON2^ cell lines

**Fig. 2:**
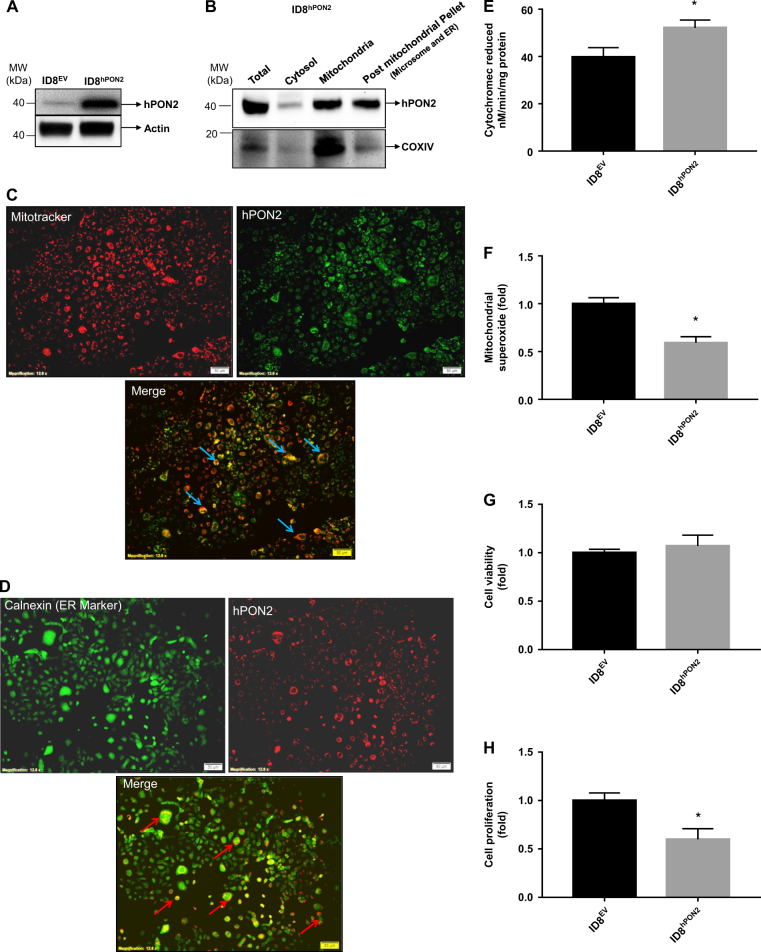
Characterization of ID8^EV^ and ID8^hPON2^ cells. **a)** Expression of hPON2 protein was analyzed by western blotting in ID8^EV^ and ID8^hPON2^ stable cell lines. **b)** ID8^EV^ and ID8^hPON2^ cell fractions were subjected to western blotting for PON2 and COX IV proteins. **c**) Colocalization of mitochondrial marker and PON2. ID8^EV^ and ID8^hPON2^ cell were fixed and stained with Mito Tracker® Red (a mitochondrial marker, red) and PON2 antibody (green). The merged image of hPON2 and mitochondria (yellow) is shown in the bottom panel. **d)** Colocalization of ER marker and PON2. ID8^EV^ and ID8^hPON2^ cell were fixed and stained for Calnexin (green), an ER marker, and hPON2 (red). The merged image of hPON2 and Calnexin is shown in the bottom panel. **e)** Mitochondria were isolated from ID8^EV^ and ID8^hPON2^ cells and respiratory complex II + III assay was carried out as described in Materials and methods section. The results are expressed as Cytochrome c reduced/min/mg protein. **f)** mitochondrial superoxide levels were measured as described in the methods  and values were expressed as fold change over the ID8^EV^. *p < 0.05 compared to ID^EV^ cells. **g)** ID8^EV^ and ID8^hPON2^ cells were cultured for 48 h, serum starved for 12 h, and cell viability was measured as described in the method section. **h**) 5 × 10^3^ ID8^EV^ and ID8^hPON2^ cells were cultured for 48 h, serum starved for 12 h and cell proliferation was assessed using BrdU by spectrophotometry as described in the Materials and methods section

To study the role of PON2 in ovarian cancer, ID8-cells were transfected with either a pcDNA 3.1 vector carrying a human PON2 cDNA (hPON2) or pcDNA 3.1 vector alone and stable cell lines (ID8^hPON2^ and ID8^EV^, respectively) were established and stable mouse ovarian cancer cell lines were generated using G418 as a selective marker. As shown in Fig. 2a, hPON2 was detected only in ID8^hPON2^ cells, but not in ID8^EV^. Subcellular fractions were isolated and PON2 was detected in mitochondria and post-mitochondrial pellet (endoplasmic reticulum and microsomal fractions) by western blotting (Fig. 2b). We further confirmed the subcellular localization of hPON2 with ID8^hPON22^ cells using immunofluorescent studies. As seen in Fig. 2c, d, hPON2 was localized to mitochondria and ER. Furthermore, functional assays demonstrated that mitochondrial ETC Complex II + III activity (Fig. 2e) was significantly increased along with decreased mitochondrial (Fig. 2f) superoxide levels in ID8^hPON2^ compared to that of ID8^EV^ cells. There was no difference in GRP78 and CHOP expression between ID8^EV^ and ID8^hPON2^ cells (data not shown). While there was no difference in cell viability between ID8^EV^ and ID8^hPON2^ cells, cell proliferation was significantly decreased in ID8^hPON2^ cells compared to ID8^EV^ cells (Fig. 2g, h).

### Expression of hPON2 reduces ID8 cell capacity to form tumors in mice

**Fig. 3:**
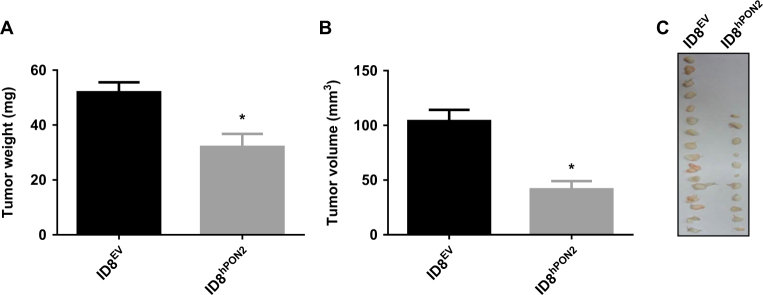
ID8^hPON2^ cell derived flank tumors have reduced tumor weight and volume. ID8^EV^ or ID8^hPON2^ cells (5 × 10^6^per mouse) were injected subcutaneously in the right flank of C57BL/6J (*n* = 15 per group). After 5 weeks, mice were sacrificed and tumors were resected and analyzed. **a**) Tumor weight was measured and expressed in mg. **b**) Tumor volumes were determined by Vernier calipers measurements (using the formula 1/2 × *L *× *W*^2^) and expressed in mm^3^. **c**) Representative tumors from the two groups of mice. Four animals did not develop visible tumors in ID8^hPON2^ group. *p < 0.05 compared to ID8^EV^-derived tumors

To test whether overexpression of hPON2 modulates tumor growth, an *in vivo* experiment was performed as described in the figure legends. As seen in Fig. 3a–c, when compared to mice that received ID8^EV^ cells, mice that received ID8^hPON2^ cells had significantly (*p *< 0.01) reduced tumor weight (ID8^EV^ mice 51 ± 14 mg vs. ID8^hPON2^ mice 32 ± 14 mg) and reduced tumor volume (ID8^EV^ 104 ± 40 mm^3^ vs. ID8^hPON2^ 41 ± 27 mm^3^).

### hPON2-mediated gene expression and cell proliferation in an *ex vivo* model

To determine the mechanism by which overexpression of hPON2 inhibits tumor size and volume, microarray-based gene expression profiling was performed on total RNA isolated from ID8^hPON2^ and ID8^EV^ cells. Detailed gene expression changes and associated pathways are shown in Supplementary Tables 1–3. In ID8^hPON2^ cells, the expression profile of many genes known to regulate tumor growth including BRCA1, cyclin and cyclin-dependent kinase 20, PTK2, Hypoxia-upregulated protein 1, p21-activated kinase 3, IL-18, IL-33, IGF-1, IGFBP2 and RNA-binding protein 9, were identified.

### PON2 expression reduces IGF-1 expression and inhibits ID-8 cell proliferation

**Fig. 4:**
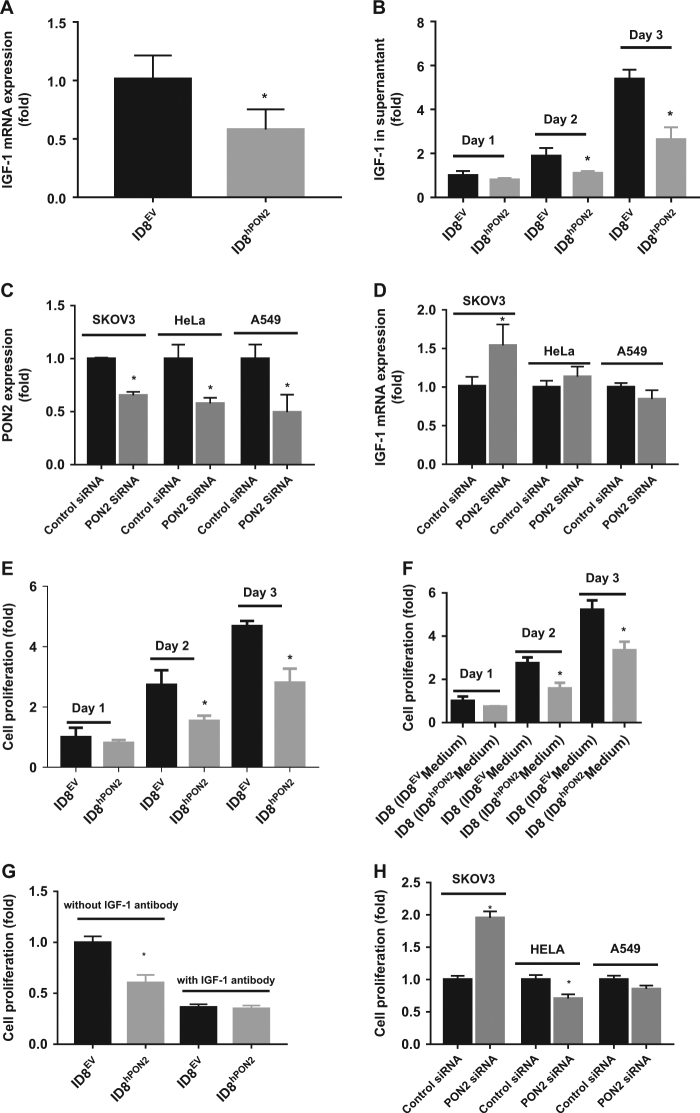
PON2 overexpression reduces IGF-1 expression and inhibits cell proliferation in ID8 cells. **a**) 3 × 10^5^ ID8^EV^ cells or ID8^hPON2^ cells were cultured on six-well plates in DMEM medium containing high glucose and l-glutamine (2 mM) and supplemented with 4% fetal bovine serum (FBS), penicillin (100 U ml^−1^), streptomycin (100 μg ml^−1^), and 1× ITS liquid media supplement (10 μg ml^−1^ insulin, 5 μg ml^−1^ transferin, and 5 ng ml^−1^ sodium selenite). Cells were incubated at 37 °C for 24 h in 5% CO_2_ and then starved for 12 h in serum free media. Total RNA was isolated and cDNA was prepared as described under materials and methods. IGF-1 mRNA was quantified by qPCR and normalized to cyclophilin. **b)** 3 × 10^5^ ID8^EV^ and ID8^hPON2^cells were grown 1–3 days and each day serum starved for 12 h, then cell culture supernatants were collected and IGF-1 protein levels were quantified by ELISA as described under Materials and methods. hPON2 siRNA or scrambled siRNA was transfected into SKOV3, HeLa, and A549 cells at 70% confluence. PON2 protein expression was quantified after 2 days as described under the Materials and methods (**c**). Total RNA was isolated and IGF-1 was quantified by qPCR (**d**). **e)** 5 × 10^3^ ID8^EV^ and ID8^hPON2^ cells were grown as indicated in **b** and cell proliferation was assessed using Brdu by spectrophotometry as described in the Materials and methods. **f**) ID8^EV^ and ID8^hPON2^ cells were grown and serum starved as in **b** and each day conditioned media were collected. Subsequently, the media were incubated with ID8 cells for 48 h, and cell proliferation was assessed as described in the Materials and methods section. **g)** ID8^EV^ and ID8^hPON2^ cells were grown for 2 days and serum starved for 12 h with 500 nM IGF-1 antibody (# ab9572, Abcam) or without IGF-1 antibody and cell proliferation was assessed as described in the materials and methods section. **h)** Following hPON2 siRNA transfection with SKOV3, HeLa, and A549 cells, cell proliferation was assessed using FITC Brdu. Brdu-positive cells were quantified using flow cytometer. *p < 0.05, compared to ID8^EV^. Values were expressed as fold (*n* = 3)

Among genes listed in Supplementary Tables 1–3, IGF-1, an inducer of cell proliferation was selected for further investigation since IGF-1 has previously been implicated in ovarian cancer^21^. We first validated IGF-1 gene expression using qPCR (Fig. 4a). IGF-1 gene expression was significantly decreased (~2.25 fold) in ID8^hPON2^ cells compared to ID8^EV^ cells (Fig. 4a). Furthermore, at protein levels  on day 1 in cell culture supernantant showed trend towards reduced IGF-1 levels in ID8^hPON2^ when compared to ID8^EV^ cells, and by day 2 and day 3, when compared to ID8^EV^ cells, IGF-1 was significantly decreased in ID8^hPON2^ cells (Fig. 4b). IGF-1 gene expression in tumor tissues and plasma IGF-1 level were significantly reduced in mice that received ID8^hPON2^ cells when compared to mice that received ID8^EV^ cells (Supplementary Fig. 1A).

We next examined the effect of endogenous PON2 inhibition on IGF-1 expression in human cancer cell lines including ovary (SKOV3), cervical (HeLa), and lung (A549). PON2 gene expression was reduced by more than 50% in all the cell types tested following transfection with PON2 Si RNA (Fig. 4c). IGF-1 expression was significantly increased in SKOV3 cells (Fig. 4d) but not in HeLa and A549 cells, suggesting that there might be a cell specific role for PON2 in mitigating IGF-1 expression. Knockdown of PON2 in ID8 cells (mouse ovarian cancer cells) also resulted in increased IGF-1 expression like that observed in SKOV3 cells (data not shown).

We examined whether hPON2 modulates cell proliferation using Brdu incorporation assays. Since FBS contains various hormones and growth factors including IGF-1, we optimized conditions for serum starvation (to synchronize cells) without completely blocking cell proliferation. We determined that 12 h starvation is ideal for these experiments (Supplementary Fig. 2). As seen in Fig. 4e, there was a trend towards reduced proliferation in ID8^hPON2^ cells when compared to ID8^EV^ cells by day 1 in an* ex vivo model*. Differences in proliferation between the ID8^EV^ and ID8^hPON2^ were significant by day 2 and day 3.

To determine whether the effect on proliferation is cell autonomous, we obtained conditioned medium (days 1, 2, and 3) from ID8^hPON2^ and ID8^EV^ cell cultures as indicated in figure legend and incubated them with ID8 cells for 24 h and cell proliferation was measured. Treating ID8 cells with conditioned medium from ID8^hPON2^ cells resulted in reduced proliferation (significant difference on day 2 and 3 but not day 1) when compared to ID8 cells treated with conditioned medium from ID8^EV^ as seen in Fig. 4f. To determine whether decreased cell proliferation in ID8^hPON2^ was due to IGF-1 levels, ID8^EV^ and ID8^hPON2^ cells were grown for 2 days and serum starved for 12 h with 500 nM IGF-1 antibody or without IGF-1 antibody and cell proliferation was assessed. In the presence of IGF-1 antibody, the proliferation of both ID8^EV^ and ID8^hPON2^ cells was inhibited and the level of proliferation was not different between the two cell types suggesting PON2 inhibits cell proliferation via IGF-1 levels (Fig. 4g). Additionally, cell proliferation was assessed with different cell types. Relative to scrambled siRNA-transfected cells, cell proliferation was significantly increased in PON2-siRNA transfected SKOV3 cells in contrast to PON2-siRNA transfected HeLa cells (Fig. 4h). However, there was no change in cell proliferation between PON2-siRNA and scrambled siRNA transfected A549 cell lines (Fig. 4h).

### PON2 reduces the IGF-1 level via C-Jun transcription factor in ID8 cells

**Fig. 5:**
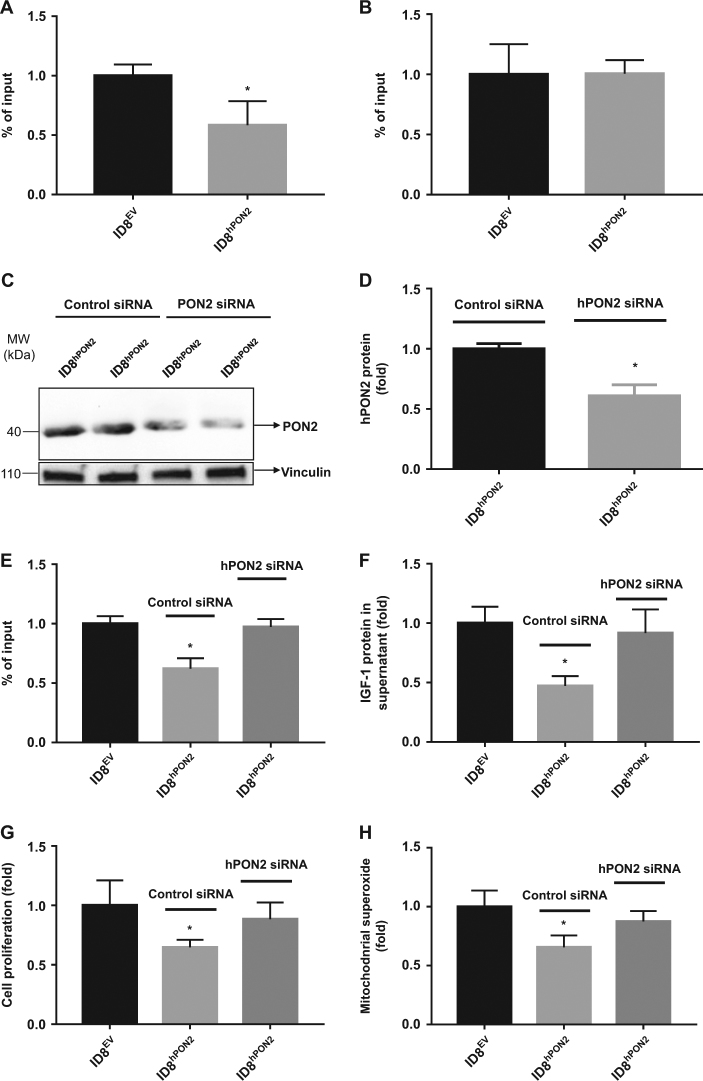
PON2 reduces the IGF-1 level via C-Jun transcription factor in ID8 cells. ID8^EV^ and ID8^hPON2^ cell culture lysates were prepared and utilized for chromatin immunoprecipitation assays with C-Jun (**a**) and p53 (**b**) antibodies. **c)** Equal number of ID8^hPON2^ cells were plated in six-well plates. At 60 % confluence level, ID8^hPON2^ cells were transfected with either hPON2 siRNA or scrambled siRNA, and PON2 expression analyzed by western blotting. **d)** Data in **c** are quantified using ImageJ software. **e)** Four six-well plates of cells were pooled from PON2-siRNA-treated ID8^hPON2^ or scrambled siRNA-treated ID8^hPON2^ cells. Chromatin immunoprecipitation assay was performed and C-Jun promoter was quantified (**e**) as described in the materials and methods section. Cell culture supernatants obtained from cells described under (**e**) and IGF-1 level was analyzed by ELISA (**f**), **g**) cell proliferation, and **h)** mitochondrial superoxide levels were measured as described in the method section. *p < 0.05, compared to ID8^EV^. Values were expressed as fold changes (*n* = 3)

Since C-Jun and P53 transcription factors induce a number genes implicated in tumor formation, we examined whether these transcription factors influence IGF-1 levels. ChIP experiments revealed that the recruitment of C-Jun (but not p53) to the IGF-1 promoter was lowered (Fig. 5a, b) in ID8^hPON2^ cells. To further, confirm whether hPON2 is directly responsible for this effect, hPON2 was silenced with siRNA in ID8^hPON2^ cells and were assayed for hPON2 protein expression, c-Jun recruitment, IGF-1 expression, cell proliferation, and mitochondrial superoxide levels. As seen Fig. 5c, d, PON2 expression was significantly reduced in siRNA-treated ID8^hPON2^ compared to scrambled siRNA-treated ID8^hPON2^ cells. Furthermore, upon PON2 siRNA treatment, the difference between ID8^EV^ and ID8^hPON2^ cells with respect to C-Jun recruitment (Fig. 5e), IGF-1 (Fig. 5f), cell proliferation (Fig. 5g), and mitochondrial superoxide (Fig. 5h) levels were significantly decreased.

### PON2 inhibits cell proliferation via IGF-1 signaling pathway by altering the caveolin-1– IGF-1R interaction in ID8 cells

**Fig. 6:**
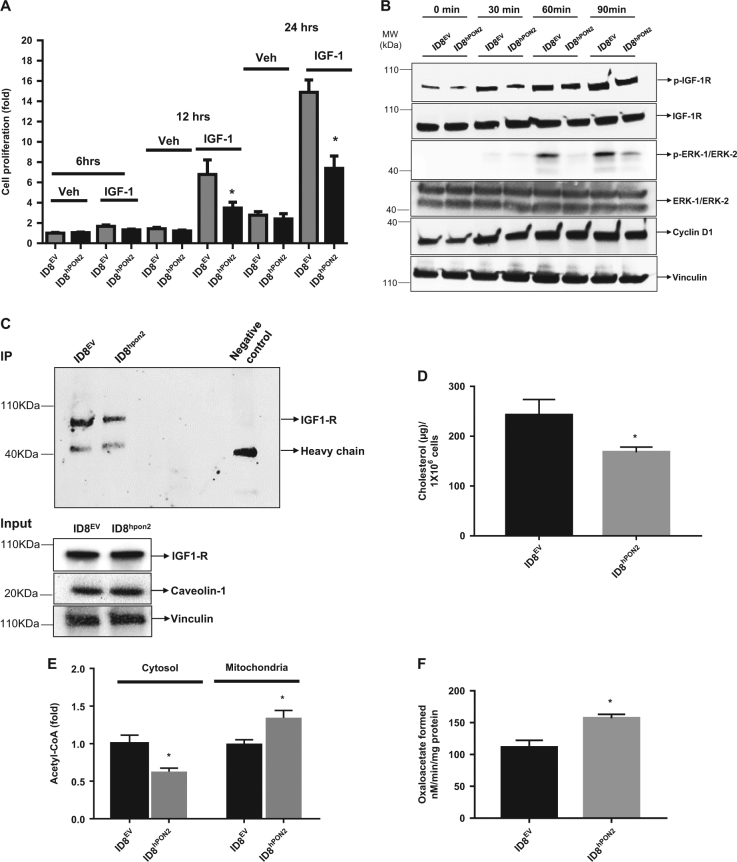
Overexpression of hPON2 inhibits cell proliferation via IGF-1 signaling pathway by altering the caveolin-1 and IGF-1R interaction in ID8 cells. **a)** 5 × 10^3^ cells were plated in 96 well plates and were incubated at 37 °C in 5% CO_2_. After 24 h, cells were serum starved for 12 h. Following this, cells were treated with IGF-1 at the concentration of 500 nM or vehicle for 6 h, 12 h, and 24 h. Cell proliferation was measured as described above. **b)** ID8^EV^ cells or ID8^hPON2^ cells were cultured in six-well plates as described above and treated with IGF-1 at the concentration of 1 µM for 30, 60, and 90 min. 50 µg of total protein (100 µg for ERK1/2) was subjected to western blotting and phosphorylated IGF-1R, total IGF-1R, phosphorylated ERK1/2, total ERK1/2, cyclin D1, and vinculin were detected using respective antibodies as described in the materials and methods section. **c**) Immunoprecipitation was performed with caveolin-1 antibody and IGF-1R was detected by western blotting. **d)** Lipids were extracted from ID8^EV^ and ID8^hPON2^ cells using chloroform: isopropanol: NP (7:11:0.11), air dried at 50 °C to remove the chloroform and placed under vacuum for 30 min, dispersed with assay buffer and cholesterol was measured as described in the Materials and methods section. **e)** Mitochondria and cytosol were isolated as described in the materials and methods section and acetyl CoA was measured using a kit according to manufacturer’s protocol. Values were normalized based on protein concentration. Values were expressed as fold change (*n* = 3). **f**) Citrate lyase enzyme activity was measured as described in the Materials and methods section. (*n *= 3). *p < 0.05, compared to ID8^EV^

To determine whether PON2 inhibits cell proliferation via IGF-1^22^ alone we performed restoration experiments as described in the figure legends (Fig. 6a). At 12 h and 24 h, cell proliferation was induced in both ID8^EV^ and ID8^hPON2^ cells. However, compared to ID8^EV^, ID8^hPON2^ cells had reduced cell proliferation (Fig. 6a). These results suggested that at early time points reduction in cell proliferation in ID8^hPON2^ cells is mediated by factors other than IGF-1 protein because IGF-1 secretion was decreased only 72 h after plating these cells. But here, even 48 h after plating (i.e., 24 h plating, 12 h serum starvation, 12 h treatment), there was decreased cell proliferation. As a first step, we examined IGF-1 signaling pathway as described in the figure legends (Fig. 6b). While IGF-1R was not altered, phosphorylation of IGF-1R gradually increased. However, compared to ID8^EV^ cells, phosphorylation of IGF-1R was lower in ID8^hPON2^ cells (Fig. 6b). Similarly, although the total level of ERK-1/ERK-2 was not different, phosphorylation of ERK-1/ERK-2 was significantly increased upon IGF-1 treatment in both groups. However, compared to ID8^EV^ cells, phosphorylation of ERK-1/ERK-2 was lower in ID8^hPON2^ cells. Same trend was seen for cyclin D (Fig. 6b).

Since deficiency of caveolin-1, a principle component of caveolae (a special type of lipid raft present in plasma membrane) decreases the IGF-1 signaling^23^, we measured caveolin-1 levels in our experiments. While there was no difference in caveolin-1 levels and total IGF-1R levels, caveolin-1–IGF-1R interaction levels were lower as seen in Fig. 6c. Since cholesterol-depleted cells disturb the structure of rafts and lead to the release of their protein components into the adjacent plasma membrane, we measured both intra- and extracellular (medium) cholesterol levels. In addition, acetyl CoA, a precursor of cholesterol biosynthesis, was measured in the mitochondria and cytosol. Relative to ID8^EV^, intracellular (Fig. 6d) and extracellular (not shown) cholesterol levels were significantly lower in ID8^hPON2^ cells. As seen in Fig. 6e, cytosolic acetyl CoA was significantly lower, whereas mitochondrial acetyl CoA significantly increased in ID8^hPON2^ cells as compared to that of ID8^EV^. Knockdown of PON2 in ID8 cells reversed this effect (data not shown). A cytosolic citrate lyase enzyme converts citrate (derived from mitochondria) into acetyl CoA, which was significantly lower (Fig. 6f) in ID8^hPON2^ cells as compared to that of ID8^EV^ cells.

**Fig. 7:**
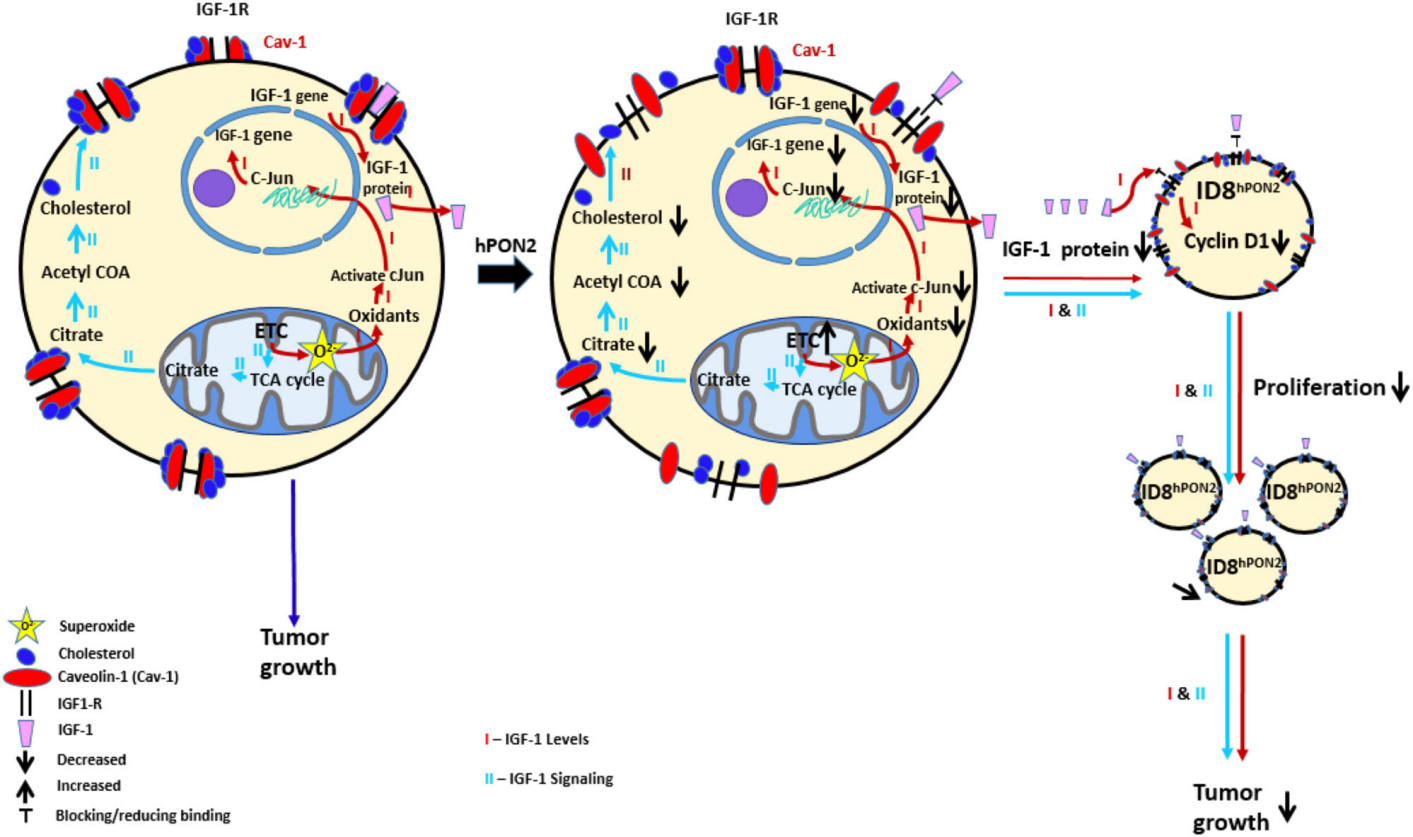
Schematic presentation of a working model for the role of PON2 in ovarian tumor growth. ID8 cells form tumors in immunocompetent C57BL6/J mice. ID8^hPON2^ cells overexpressing human PON2 develop reduced tumors. Based on the data presented in this paper, PON2 overexpression regulates three anti-tumorigenic pathways. I). PON2 overexpression reduces mitochondrial superoxide levels, which regulate c-Jun activation. Reduced c-JUN binding to the IGF-1 promoter leads to decreased expression of IGF-1 protein. II). PON2 expression enhances electron transport chain activity leading to decreased cholesterol levels resulting in impaired caveolin-1 and IGF-1R interaction. I & II result in decreased cell proliferation and reduced tumor growth

To ascertain that our results are not ID8 cell specific, we confirmed key results described above in an additional ovarian cancer cell line OVCAR-5 cells (Supplementary Results and Supplementary Fig. 3). Figure 7 illustrates the anti-tumorigenic effects of hPON2 in ovarian cancer.

## Discussion

Despite growing evidence suggesting that PON2 ameliorates atherosclerosis, Type II diabetes, and neurodegenerative diseases^9,13,23^, its role in cancer has not been established in an *in vivo* model. In this study, using an ovarian cancer model, we report, for the first time, that: (1) PON2 is overexpressed in early stages (but not in late stages) of ovarian cancer and (2) in mice, overexpression of PON2 reduces ovarian tumor size and volume in a xenograft model. Our results also demonstrate novel mechanisms for PON2 involving IGF-1 expression and signaling and regulation of immune function.

### Why PON2 is differentially regulated at various stages of ovarian cancer and what is its role in ovarian tumor formation?

It has been reported that PON2 and PON3 are upregulated in various cancer tissues including ovarian cancer^14,15,19,24–28^. Studies suggest that PON1 polymorphisms are associated with various cancers including ovarian cancer and its activity was negatively correlated with ovarian tumor size in human subjects providing clinical evidence for a link between PON and cancer^29–31^. This is the first report demonstrating a role of PONs in an in vivo cancer model. This present study suggests that PON2 is overexpressed in early stages of ovarian cancer in human subjects (Fig. 1a, b) and its expression is negatively associated with tumor size and volume (Fig. 3a–c) in a mouse model. Senthil *et al*.^32^ reported that plasma from ovarian cancer patients shows decreased anti-oxidant enzyme activities including catalase, extracellular SOD, and non-enzymatic antioxidants such as vitamin C and E along with increased concentrations of plasma thiobarbituric acid reactive substances and conjugated dienes. Intracellular ROS may induce PON2 expression and our in vivo model further implies that induction of PON2 as a protective mechanism. Prior studies suggest that PON2 is upregulated in response to oxidative stress in both an *ex vivo* and* in vivo* model^33,34^. Consistent with our study, Yumin* et al*.^35^ have reported that mitochondrial Mn superoxide dismutase (Mn SOD) (which converts toxic superoxide to the less toxic hydrogen peroxide) expression was high in ovarian tumor tissue and its expression was induced by mitochondrial oxidative stress. Over expression of mitochondrial Mn-SOD reduced ovarian cancer tumor growth by ameliorating mitochondrial superoxide/oxidative stress^35^. Balliet *et al.*^36^ demonstrated that mitochondrial transcription factors A (TFAM) deficient fibroblasts showed evidence of mitochondrial oxidative stress, decreased respiratory complex activity, with the loss of certain mitochondrial respiratory chain components and significantly promoted the tumor growth in a breast cancer xenograft model. Although currently it is unknown why PON2 was downregulated in late stages of ovarian cancer compared to early stages within the ovary and in lymph node where it was increased. Possibilities include (1) diverse cell–cell interactions in early vs. late stages that lead to different PON2 expression and (2) adaptive mutations in late stage that prevent PON2 expression. Whatever the cause, based on our i*n vivo model,* we can conclude that decreased expression of PON2 in late stages would be associated with cancer progression. Further work needs to address the role of PON2 in an *in vivo* metastatic tumor model.

### How does PON2 reduce IGF-1 levels and cell proliferation in ovarian cancer cell lines?

Perturbation of redox homeostasis alters c-Jun activation. For example, activated forms of c-Jun and JNK have been reported in muscle fibers with respiratory chain deficiency along with increased mitochondrial ROS^37^. Further, Li *et al*. reported that embryonic fibroblasts from c-Jun knockout mice show decreased secretion of IGF-1 and inhibited benign prostatic hyperplasia-1 proliferation^38^. Mice lacking c-Jun in hepatocytes had reduced IGF-1 expression^39^. Our ChIP study suggests that decreased mitochondrial superoxide levels (Fig. 2f) in ID8^hPON2^ recruit c-Jun at the promoter of IGF-1 to a lesser degree (Fig. 5a). The present study demonstrates that mitochondrial superoxide was significantly decreased in ID8^hPON2^ cells, but there was no difference in ER stress marker genes such as GRP78 and CHOP between ID8^EV^ and ID8^hPON2^ suggesting mitochondrial ROS plays an important role in activating the c-Jun transcription factor, which further regulates IGF-1 levels.

Using various cancer cell line models, it has been shown that overexpression of PON2 protects from cell death by reducing mitochondrial superoxide levels. Based on this information, Witte et al. speculated that PON2 may promote tumor growth^40^. It should be noted that cancer is a multistage process of initiation, progression, and metastasis. Superoxide/oxidative stress not only induces apoptosis, it also can stimulate the epithelial–mesenchymal transition processes where epithelial cells alter various metabolic processes that permit development of a mesenchymal phenotype, a process that is responsible for initiation and metastasis^41,42^. In addition, it has been shown that ROS/antioxidant genes modulate tumorigenesis in a spatiotemporal manner. For example, NRF2 a transcription factor (that induces antioxidant gene expression) inhibits cell proliferation in U25MG human glioma cells and tumor growth in a xenograft mouse model^43^ but it induces cell proliferation in human hepatocellular carcinoma lines^44^. Based on specific requirements and cellular chemistry, ROS modulates the survival pathway or death pathway by altering gene expression/activity with different cell types.

### How does PON2 reduce IGF-1 signaling in ovarian cancer cells?

Receptor and ligand levels cannot solely explain receptor autophosphorylation and its downstream signaling pathways. For example, in type II diabetes, despite insulin and insulin receptor-1 (IRS-1) levels being similar to control subjects, the signaling pathway is impaired^45^. Accumulating evidence suggests that membrane fluidity, clustering of the receptors, and receptors and other protein interactions that define signaling pathways are mediated by cellular cholesterol levels^46^. The precursor of cholesterol is acetyl CoA which is mainly generated from fatty acid oxidation and pyruvate decarboxylation processes both of which occur in the mitochondria. Since acetyl CoA cannot enter the cytosol, an intermediate compound of the tricarboxylic acid (TCA) cycle, “citrate” exits the mitochondria to enter the cytosol where it reacts with Co-A to form acetyl-CoA and oxaloacetate by citrate lyase^47^. Furthermore, electron transport chain (ETC) and the TCA cycle are tightly regulated^48^. For example, ETC generates the NAD and FAD which act as cofactors in the TCA cycle^48^. Thus, decreased cholesterol in ID8^hPON2^ (Fig. 6d) cells may be due to constantly increased ETC activity triggering the TCA cycle instead of forming acetyl CoA from citrate. Indeed, in our study, succinate dehydrogenase (the only enzyme involved in TCA cycle ETC) activity was increased (Fig. 2e) in PON2-overexpressing cells with decreased acetyl CoA (Fig. 6e) in the cytosol. Previously, it has been shown that PON2 deficiency in mice increases the serum cholesterol level^49^. Macrophage cholesterol biosynthesis and PON2 expression was negatively correlated in Delta6-Desaturase knockout mice^50^. Despite decreased cholesterol in ID8^hPON2^ cells, plasma cholesterol was not significantly changed in mice receiving ID8^hPON2^ compared to ID8^EV^. In the current study, neither caveolin-1 nor IGF-1R protein levels were changed (Fig. 6c), but the interaction was impaired (Fig. 6c). It has been reported that depleting cholesterol in cells or sequestering cholesterol from the membrane causes a shift in raft and caveolar proteins and hormonal receptors to other parts of the plasma membrane^46^. It has been reported that cavolin-1 regulates the IGF-1R signaling in H9C2 cells, rat cardio myoblasts and adipose tissue^51^. Hormonal receptor-caveolin-1 interaction is cell specific. For instance, The interaction of insulin receptor–cavolin-1 influences IRS-1 in COS-7 cells but not adipose cells, upon insulin binding^52^

### Why is PON2 a good target for ovarian cancer therapy?

Clinical studies suggest a role for IGF-1 in ovarian cancer etiology. For example: (1) a prospective study from Lukanova *et al.*^53^ demonstrated that there was a positive correlation between circulating IGF-I levels and the risk of developing ovarian cancer. (2) Brokaw *et al*.^54^ reported that women with high IGF-I mRNA and IGF-1 peptide levels were at a greater risk for disease progression compared to those with low levels, suggesting that IGF-I is involved in ovarian cancer progression. (3) Altered expression of IGF axis member genes was associated with poor prognosis in epithelial ovarian cancer^55^. Several small molecule tyrosine kinase inhibitors against IGF-1R inhibited tumor growth *in vivo* model^56^. However, because of the high degree of homology between IGF-1R and insulin receptor (IR), most specific IGF-1R tyrosine kinase inhibitors (TKIs) have inhibitory effects on the IR^57^. For instance, OSI-906, has a half maximal inhibitory concentration (IC_50_) of 0.018 µmol L^−1^ and 0.054 µmol L^−1^ against IGF-1R and IR, respectively^57^. It is documented that impaired IR signaling induces hyperglycemia and toxicity^58^. Hence, instead of directly targeting IGF-1/IGF-1R, which has adverse effect on glucose hemostasis, activating PON2 would be a fruitful alternative strategy for treating ovarian cancer. Altogether, our study demonstrated for the first time that PON2 inhibits ovarian tumor growth.

## Electronic supplementary material

Supplemental result and legends(DOC 32 kb)

Supplementary Figures(PDF 239 kb)

Supplementary Table 1(PDF 32 kb)

Supplementary Table 2(PDF 82 kb)

Supplementary Table 3(PDF 55 kb)

## References

[1] Liou GY, Storz P (2010). Reactive oxygen species in cancer. Free Radic. Res..

[2] Zong WX, Rabinowitz JD, White E (2016). Mitochondria and cancer. Mol. Cell.

[3] Yang Y (2016). Mitochondria and mitochondrial ROS in cancer: novel targets for anticancer therapy. J. Cell. Physiol..

[4] Ness RB, Cottreau C (1999). Possible role of ovarian epithelial inflammation in ovarian cancer. J. Natl Cancer Inst..

[5] Hu Y (2005). Mitochondrial manganese-superoxide dismutase expression in ovarian cancer: role in cell proliferation and response to oxidative stress. J. Biol. Chem..

[6] Reddy ST, Devarajan A, Bourquard N, Shih D, Fogelman AM (2008). Is it just paraoxonase 1 or are other members of the paraoxonase gene family implicated in atherosclerosis?. Curr. Opin. Lipidol..

[7] Ng CJ (2005). The paraoxonase gene family and atherosclerosis. Free Radic. Biol. Med..

[8] Devarajan A (2011). Paraoxonase 2 deficiency alters mitochondrial function and exacerbates the development of atherosclerosis. Antioxid. Redox Signal..

[9] Bourquard N, Ng CJ, Reddy ST (2011). Impaired hepatic insulin signalling in PON2-deficient mice: a novel role for the PON2/apoE axis on the macrophage inflammatory response. Biochem. J..

[10] Parsanejad M (2014). DJ-1 interacts with and regulates paraoxonase-2, an enzyme critical for neuronal survival in response to oxidative stress. PLoS ONE.

[11] Costa LG (2014). Paraoxonase-2 (PON2) in brain and its potential role in neuroprotection. Neurotoxicology.

[12] Costa LG (2013). Modulation of paraoxonase 2 (PON2) in mouse brain by the polyphenol quercetin: a mechanism of neuroprotection?. Neurochem. Res..

[13] Giordano G, Cole TB, Furlong CE, Costa LG (2011). Paraoxonase 2 (PON2) in the mouse central nervous system: a neuroprotective role?. Toxicol. Appl. Pharmacol..

[14] Witte, I., Foerstermann, U., Devarajan, A., Reddy, S. T. & Horke, S. Protectors or traitors: the roles of PON2 and PON3 in atherosclerosis and cancer. *J. Lipids* **2012**, 342–806 (2012). 10.1155/2012/342806 PMC3361228 22666600

[15] Schweikert EM (2012). PON3 is upregulated in cancer tissues and protects against mitochondrial superoxide-mediated cell death. Cell Death Differ..

[16] Devarajan A (2013). Role of PON2 in innate immune response in an acute infection model. Mol. Genet. Metab..

[17] Devarajan A (2012). Macrophage paraoxonase 2 regulates calcium homeostasis and cell survival under endoplasmic reticulum stress conditions and is sufficient to prevent the development of aggravated atherosclerosis in paraoxonase 2 deficiency/apoE(-/-) mice on a western diet. Mol. Genet. Metab..

[18] Devarajan A (2012). Macrophage paraoxonase 2 regulates calcium homeostasis and cell survival under endoplasmic reticulum stress conditions and is sufficient to prevent the development of aggravated atherosclerosis in paraoxonase 2 deficiency/apoE-/- mice on a western diet. Mol. Genet. Metab..

[19] Witte I (2011). Beyond reduction of atherosclerosis: PON2 provides apoptosis resistance and stabilizes tumor cells. Cell Death Dis..

[20] Horke S (2007). Paraoxonase-2 reduces oxidative stress in vascular cells and decreases endoplasmic reticulum stress-induced caspase activation. Circulation.

[21] Brokaw J (2007). IGF-I in epithelial ovarian cancer and its role in disease progression. Growth Factors.

[22] Ukaji T, Umezawa K (2015). Inhibition of IGF-1 mediated cellular migration and invasion by migracin A in ovarian clear cell carcinoma cells. Int. J. Gynecol. Cancer.

[23] Devarajan A (2013). Role of PON2 in innate immune response in an acute infection model. Mol. Genet. Metab..

[24] Kruger M, Pabst AM, Al-Nawas B, Horke S, Moergel M (2015). Paraoxonase-2 (PON2) protects oral squamous cell cancer cells against irradiation-induced apoptosis. J. Cancer Res. Clin. Oncol..

[25] Ribarska T, Ingenwerth M, Goering W, Engers R, Schulz WA (2010). Epigenetic inactivation of the placentally imprinted tumor suppressor gene TFPI2 in prostate carcinoma. Cancer Genom. Proteom..

[26] Ross ME (2003). Classification of pediatric acute lymphoblastic leukemia by gene expression profiling. Blood.

[27] Kang H (2010). Gene expression classifiers for relapse-free survival and minimal residual disease improve risk classification and outcome prediction in pediatric B-precursor acute lymphoblastic leukemia. Blood.

[28] Frank O (2006). Gene expression signature of primary imatinib-resistant chronic myeloid leukemia patients. Leukemia.

[29] Michalak S, Szubert S, Moszynski R, Sajdak S, Szpurek D (2014). Serum arylesterase and paraoxonase activities in patients with ovarian tumors. Taiwan J. Obstet. Gynecol..

[30] Aksoy-Sagirli P (2011). Paraoxonase-1 192/55 polymorphisms and the risk of lung cancer in a Turkish population. Anticancer Res..

[31] Arpaci A, Gormus U, Dalan B, Berkman S, Isbir T (2009). Investigation of PON1 192 and PON1 55 polymorphisms in ovarian cancer patients in Turkish population. In Vivo.

[32] Senthil K, Aranganathan S, Nahni N (2004). Evidence of oxidative stress in the circulation of ovarian cancer patients. Clin. Chim. Acta.

[33] Kim JB (2011). Paraoxonase-2 modulates stress response of endothelial cells to oxidized phospholipids and a bacterial quorum-sensing molecule. Arterioscler. Thromb. Vasc. Biol..

[34] Reddy ST, Devarajan A, Bourquard N, Shih D, Fogelman AM (2008). Is it just paraoxonase 1 or are other members of the paraoxonase gene family implicated in atherosclerosis?. Curr. Opin. Lipidol..

[35] Hu YM (2005). Mitochondrial manganese-superoxide dismutase expression in ovarian cancer. J. Biol. Chem..

[36] Balliet RM (2011). Mitochondrial oxidative stress in cancer-associated fibroblasts drives lactate production, promoting breast cancer tumor growth understanding the aging and cancer connection. Cell Cycle.

[37] Filosto M (2003). Transcription factors c-Jun/activator protein-1 and nuclear factor-kappa B in oxidative stress response in mitochondrial diseases. Neuropathol. Appl. Neurobiol..

[38] Li WH, Wu CL, Febbo PG, Olumi AF (2007). Stromally expressed c-Jun regulates proliferation of prostate epithelial cells. Am. J. Pathol..

[39] Trierweiler C, Blum HE, Hasselblatt P (2012). The transcription factor c-Jun protects against liver damage following activated beta-catenin signaling. PLoS ONE.

[40] Witte I (2011). Beyond reduction of atherosclerosis: PON2 provides apoptosis resistance and stabilizes tumor cells. Cell Death Dis..

[41] Radisky DC (2005). Epithelial-mesenchymal transition. J. Cell Sci..

[42] Cichon MA, Radisky DC (2014). ROS-induced epithelial-mesenchymal transition in mammary epithelial cells is mediated by NF-kappa B-dependent activation of Snail. Oncotarget.

[43] Ji XJ (2013). Knockdown of NF-E2-related factor 2 inhibits the proliferation and growth of U251MG human glioma cells in a mouse xenograft model. Oncol. Rep..

[44] Zhang MX (2015). Nrf2 is a potential prognostic marker and promotes proliferation and invasion in human hepatocellular carcinoma. BMC Cancer.

[45] Sah SP, Singh B, Choudhary S, Kumar A (2016). Animal models of insulin resistance: a review. Pharmacol. Rep..

[46] Goluszko P, Nowicki B (2005). Membrane cholesterol: a crucial molecule affecting interactions of microbial pathogens with mammalian cells. Infect. Immun..

[47] Lemus HN, Mendivil CO (2015). Adenosine triphosphate citrate lyase: emerging target in the treatment of dyslipidemia. J. Clin. Lipidol..

[48] Desideri E, Vegliante R, Ciriolo MR (2015). Mitochondrial dysfunctions in cancer: genetic defects and oncogenic signaling impinging on TCA cycle activity. Cancer Lett..

[49] Bourquard N, Ng CJ, Reddy ST (2011). Impaired hepatic insulin signalling in PON2-deficient mice: a novel role for the PON2/apoE axis on the macrophage inflammatory response. Biochem. J..

[50] Rosenblat M, Volkova N, Roqueta-Rivera M, Nakamura MT, Aviram M (2010). Increased macrophage cholesterol biosynthesis and decreased cellular paraoxonase 2 (PON2) expression in Delta 6-desaturase knockout (6-DS KO) mice: beneficial effects of arachidonic acid. Atherosclerosis.

[51] Salani B, Briatore L, Garibaldi S, Cordera R, Maggi D (2008). Caveolin-1 down-regulation inhibits insulin-like growth factor-I receptor signal transduction in H9C2 rat cardiomyoblasts. Endocrinology.

[52] Nystrom FH, Chen H, Cong LN, Li YH, Quon MJ (1999). Caveolin-1 interacts with the insulin receptor and can differentially modulate insulin signaling in transfected Cos-7 cells and rat adipose cells. Mol. Endocrinol..

[53] Lukanova A (2002). Circulating levels of insulin-like growth factor-I and risk of ovarian cancer. Int. J. Cancer.

[54] Brokaw J (2007). IGF-I in epithelial ovarian cancer and its role in disease progression. Growth Factors.

[55] Spentzos D (2007). IGF axis gene expression patterns are prognostic of survival in epithelial ovarian cancer. Endocr. Relat. Cancer.

[56] Carboni JM (2009). BMS-754807, a small molecule inhibitor of insulin-like growth factor-1R/IR. Mol. Cancer Ther..

[57] Chen HX, Sharon E (2013). IGF-1R as an anti-cancer target-trials and tribulations. Chin. J. Cancer.

[58] Janssen JAMJL, Varewijck AJ (2014). IGF-IR targeted therapy: past, present and future. Front. Endocrinol..

