# Supplementary material for: Paraoxonase 2 overexpression inhibits tumor development in a mouse model of ovarian cancer
Source: Cell Death Dis. 2018 Mar 12;9(3):392. doi: 10.1038/s41419-018-0395-2 (PMC5847560; doi:10.1038/s41419-018-0395-2)
Supplement: Supplementary file 1 — Supplemental result and legends(DOC 32 kb) [file 41419_2018_395_MOESM1_ESM.doc]

**Supplemental Results**

**Overexpression of hPON2 in OVCAR-5 cells reduces IGF1 levels, IGF-1 signaling, cell proliferation, and improves mitochondrial function**

OVCAR-5 cells were transiently transfected with either a pcDNA 3.1 vector carrying a human PON2 cDNA (hPON2) or pcDNA 3.1 vector alone. Following two days of transfection, hPON2 expression was significantly increased along with decreased IGF-1 expression (Sup Fig. 3A-B) in OVCAR-5^hPON2^ cells when compared to OVCAR-5^EV^ cells. Cell proliferation significantly decreased in OVCAR5^hPON2^ cells compared to OVCAR-5^EV^ cells (Sup Fig. 3C-F). Following 2 days of transfection, cells were treated with fresh serum free medium containing IGF-1 at a final concentration of 5 µM for 30 minutes or 60 minutes, and IGF-1 signaling was examined by Western blotting. There was no difference in IGF-1R between two groups upon IGF-1 treatment. Although phosphorylation of IGF-1R increased in both groups, phosphorylation of IGF-1R was lower in OVCAR-5^hPON2^ cells compared to OCAR-5^EV^ cells (Sup Fig. 3G). Similar results were obtained for ERK-1/ERK-2 phosphorylation and cyclin-D1 (Sup Fig. 3G). Compared to OVCAR-5^EV^ cells, intracellular cholesterol levels (Sup Fig. 3H) were significantly lowered in OVCAR-5^hPON2^ cells. Furthermore, functional assays revealed that mitochondrial ETC Complex II + III activity (Sup Fig. 3I) was significantly increased along with decreased mitochondrial superoxide levels (Sup Fig. 3J) in OVCAR-5^hPON2^ compared to that of OVCAR5^EV^ cells. *p < 0.05, compared to OVCAR-5^EV^ cells.

**Supplemental Tables**

**Supplemental Table 1.** **Microarray profiling of ID8^EV^** **and ID8^hPON2^ cells.** 30,000 ID8^EV^ cells or ID8^hPON2^ cells were cultured on 6 well plates in DMEM medium containing high glucose and L-glutamine (2 mM) and supplemented with 4% fetal bovine serum (FBS), penicillin (100 Uml^−1^), streptomycin (100 μgml^−1^), and 1 × ITS liquid media supplement (10 μgml^−1^ insulin, 5 μgml^−1^ transferin, and 5 ngml^−1^ sodium selenite). Cells were incubated at 37ºC for 24 hrs in 5% CO_2_ and then serum starved for 12 hours in serum free media. Total RNA was isolated and microarray experiments were performed as described under materials and methods. Genes represented in the table are based on a p-value less than 0.05 and a fold change greater than or equal to 1.5 or less than or equal to 1.5 between the two groups.

**Supplemental Table 2. Altered canonical pathways between ID8^EV^ and ID8^hPON2^ cells.** Genes under Table 1 were analyzed for canonical pathways. Values are expressed -logP value.

**Supplemental Table 3.** Canonical pathways that are directly implicated in tumor biology. Values are expressed -logP value.

**Supplemental Figure Legends**

**Supplemental Figure 1. IGF-1 levels in tumors and Plasma:** (A) Total RNA was isolated from tumor tissue, cDNA was prepared, and IGF-1 gene expression was quantified using QPCR and normalized to cyclophillin. Values are expressed as fold ID8^EV^ (n=5). (B) Plasma IGF-1 protein level was quantified as described in the methods. Values are expressed as ng/mL (ID8^EV^ n = 7, ID8^hPON2^ n=9). *p < 0.05 compared to ID8^EV^ derived tumors or plasma.

**Supplemental Figure 2. Cell cycle analyses in ID8 cells:**  ID8 cells were grown, serum starved for 12 hours, 24 hours, and 48 hours. Following this, these cells were treated with propidium iodide solution and cell cycle was analyzed with flow cytometer (A) G1 phase (B) G2 Phase (C) S phase (n=3).

**Supplemental Figure 3. Overexpression of hPON2 in OVCAR-5 cells reduces IGF1 level, IGF-1 signaling, cell proliferation, and improves mitochondrial function.** OVCAR-5 cells at about 60% confluence were transfected with pcDNA 3.1 vector carrying a human PON2 cDNA (hPON2) or pcDNA 3.1 vector alone using the Lipofectamine 2000 kit (Invitrogen). Following two days of transfection, total RNA was isolated and cDNA was prepared. PON2 (A) and IGF-1 (B) were measured using QPCR and normalized to cyclophillin. Values are expressed as fold changes. Cell proliferation was carried out using flow cytometer as described in the method section, values are expressed as fold changes (C) and results are represented (D-F). (G) Following two day of transfection, cells were serum first starved for 12 hrs. Cells were then treated with IGF-1 at 5 μM for 30 min or 60 mins. Proteins were extracted with lysate buffer and 50 ug of protein (100 ug for ERK and p-ERK detection) was loaded onto the gel, then transferred to nitrocellulose membrane. Phosphorylation of IGF-1 receptor, total IGF-1 receptor, phosphorylation of ERK, total ERK1/2, cyclin D and vinculin, as an internal control were detected using respective antibodies as described in the methods section. (H) Intracellular cholesterol was measured as described in the method sections and values were expressed µg/1x10^6^ cells. (I) Mitochondria was isolated and complex II + III activity was carried out as described in the method section. (J) Mitochondrial superoxide was measured as described method section. Values are expressed as fold change. n=3. *p < 0.05 compared to OVCAR-5^EV^.
