# Supplementary figures and images for: Paraoxonase 2 overexpression inhibits tumor development in a mouse model of ovarian cancer
Source: Cell Death Dis. 2018 Mar 12;9(3):392. doi: 10.1038/s41419-018-0395-2 (PMC5847560; doi:10.1038/s41419-018-0395-2)

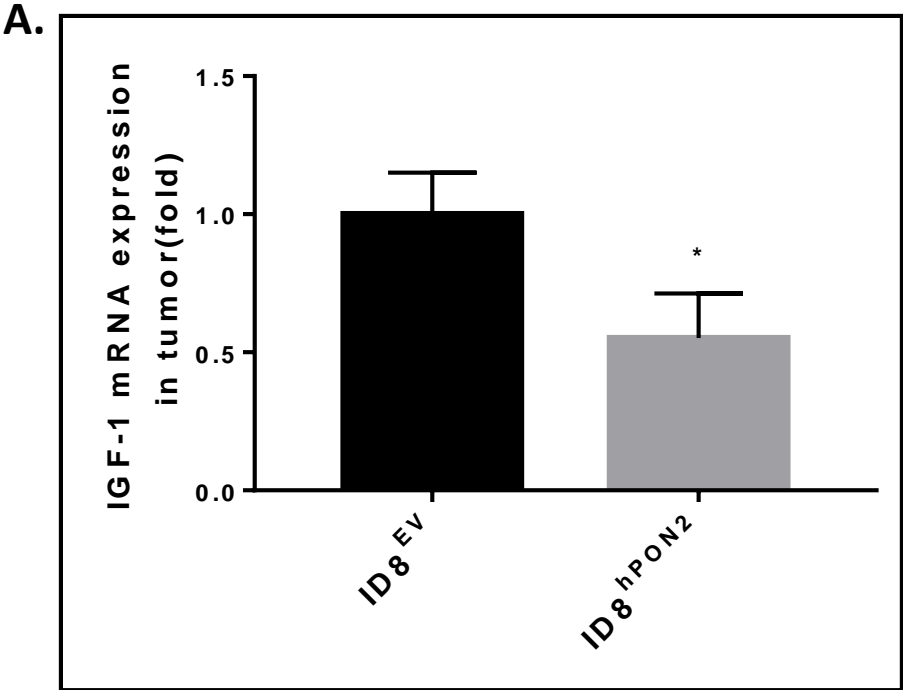

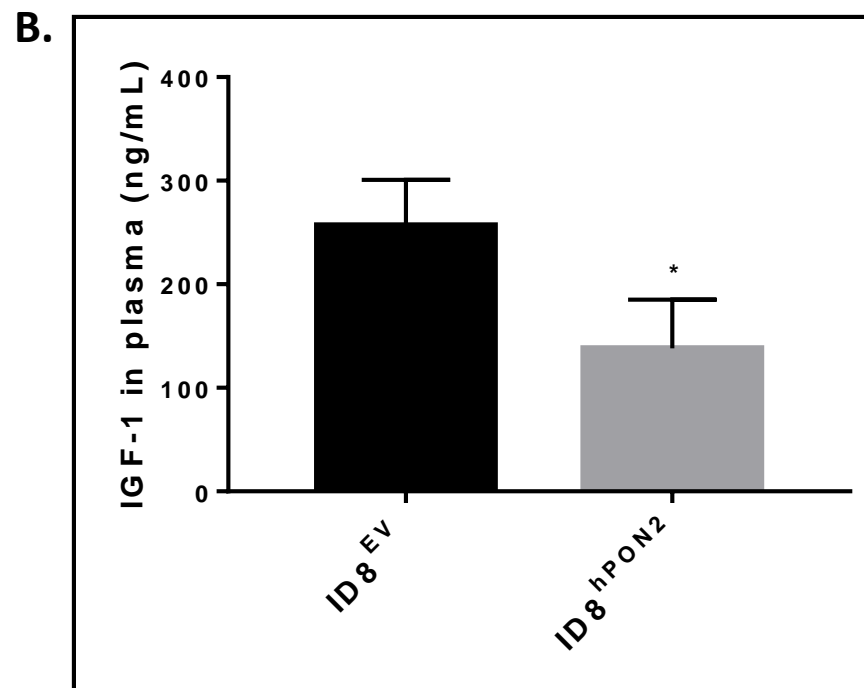

Supplementary figure 2S

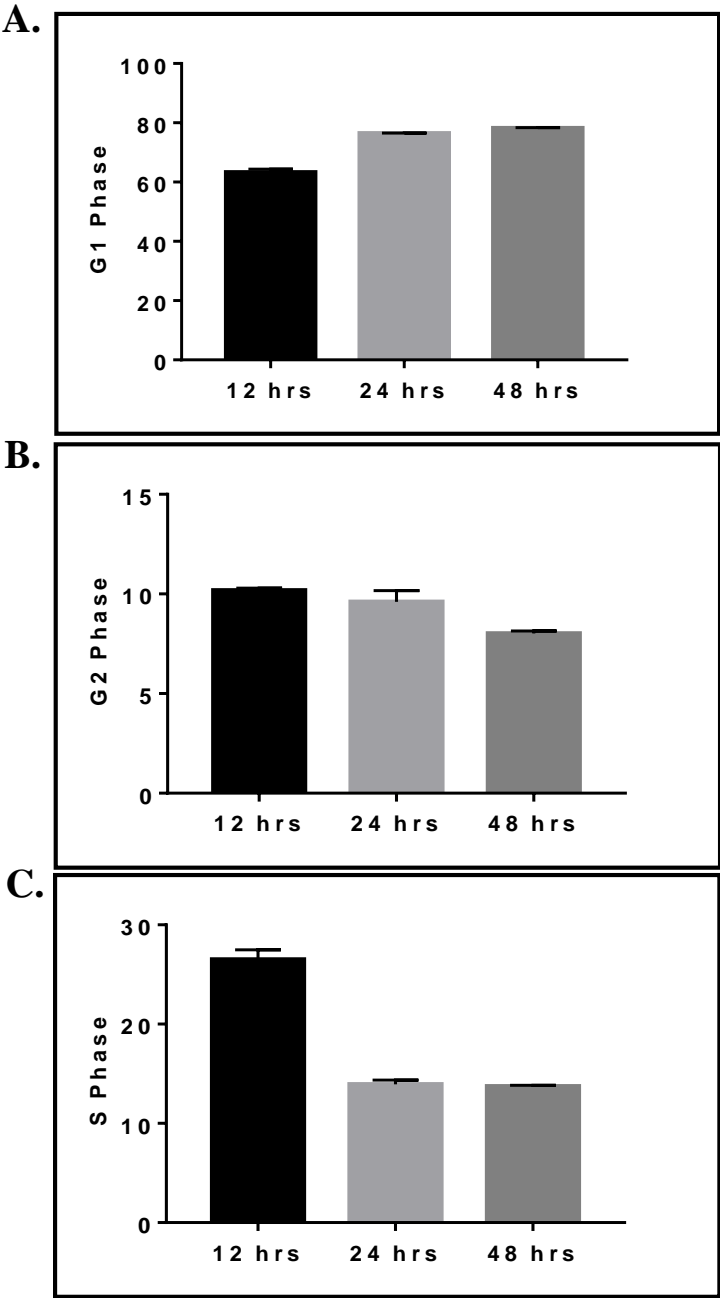

A.

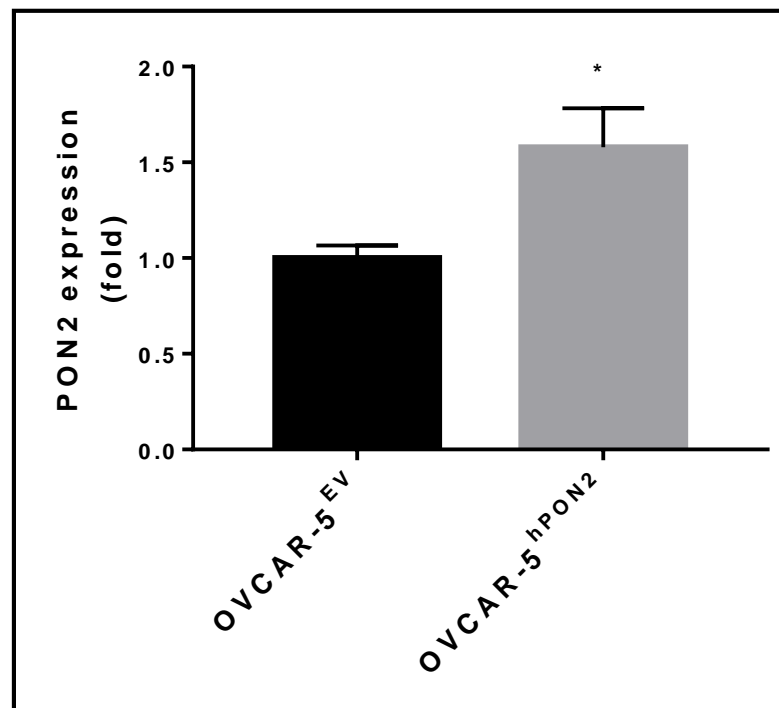

B.

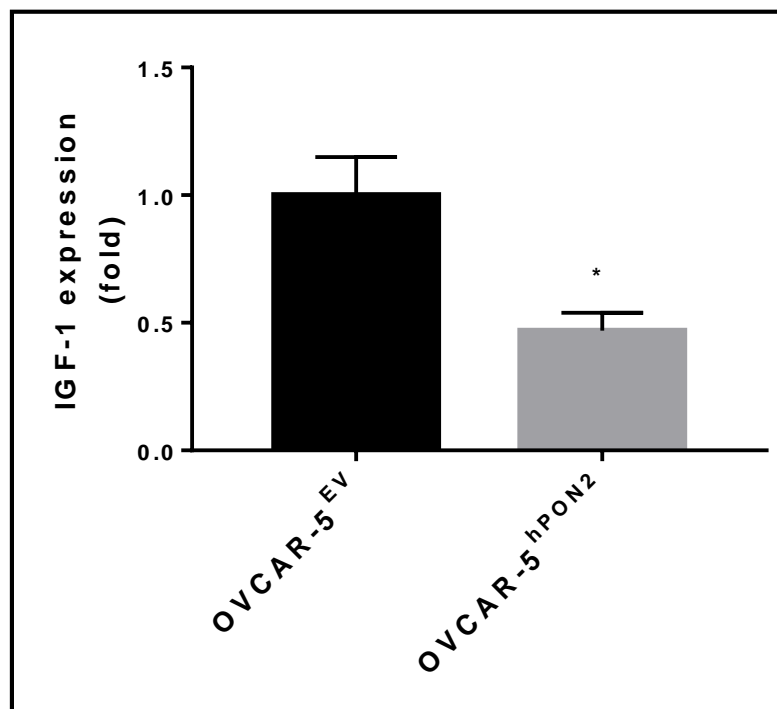

Supplementary figure 3S

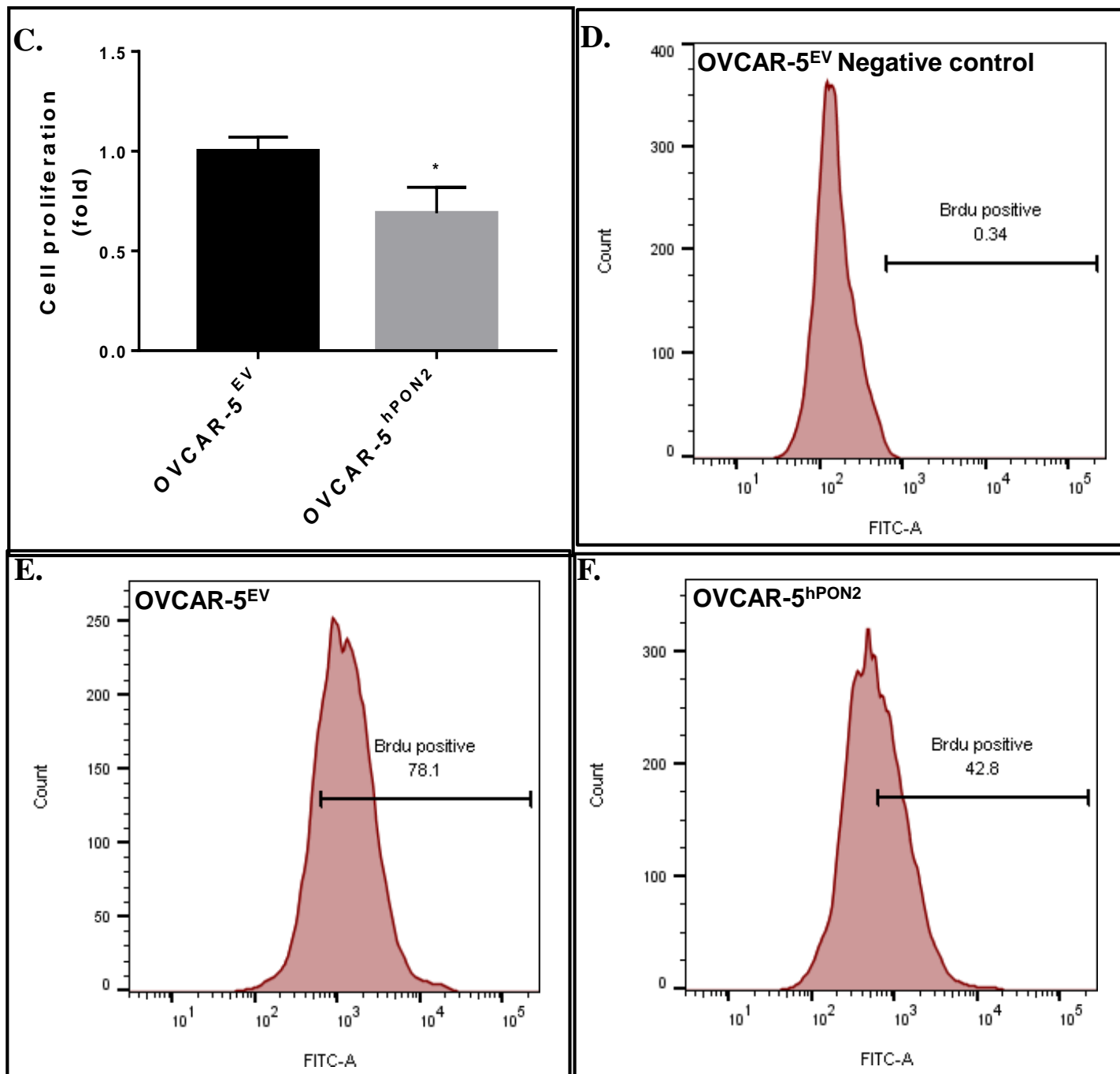

G.

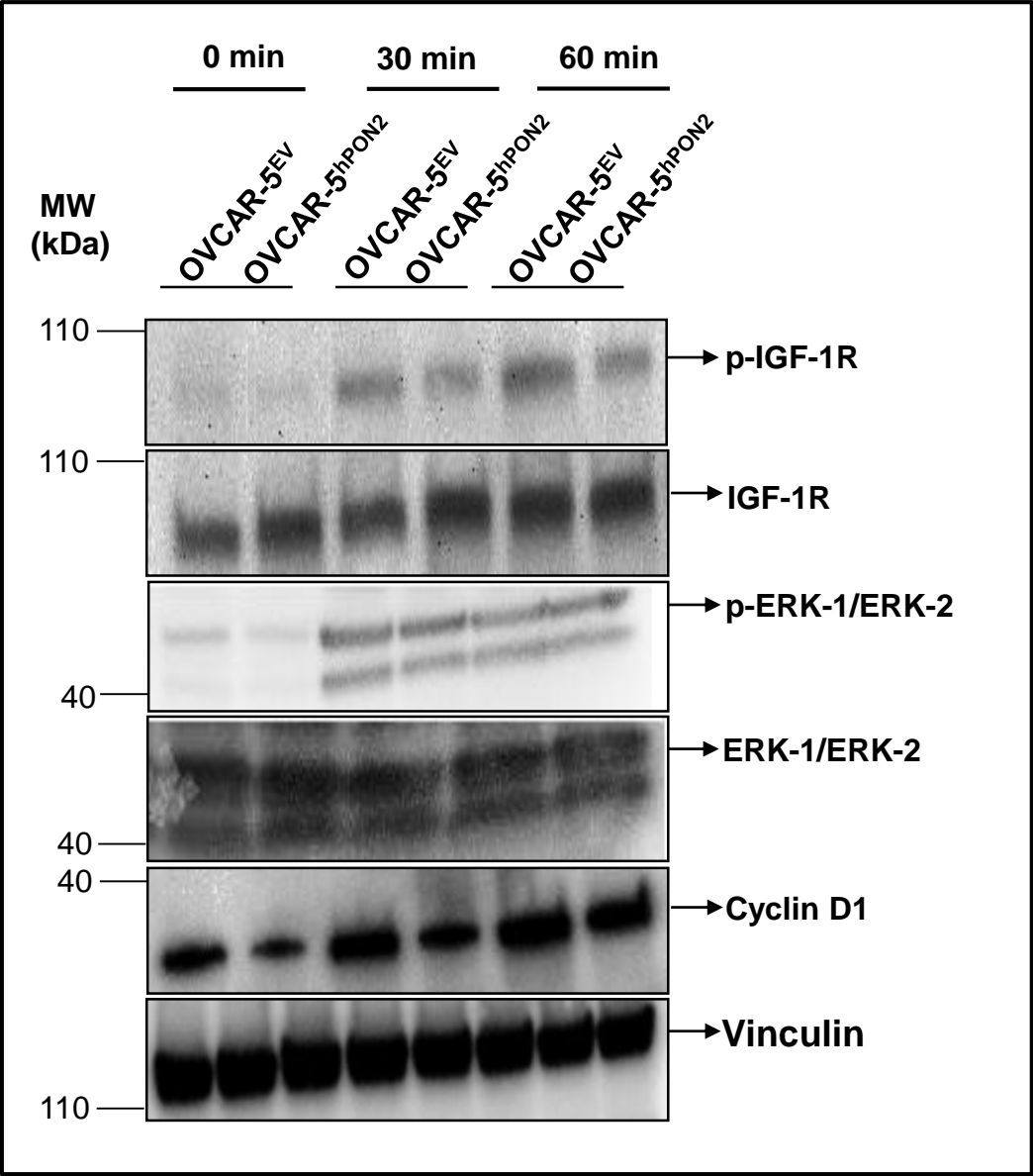

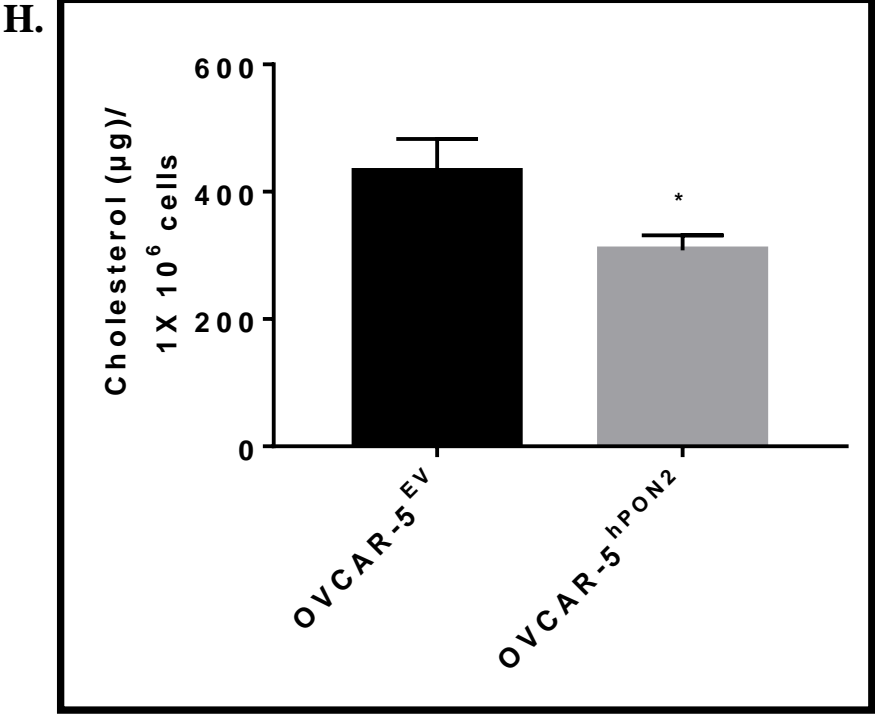

I.

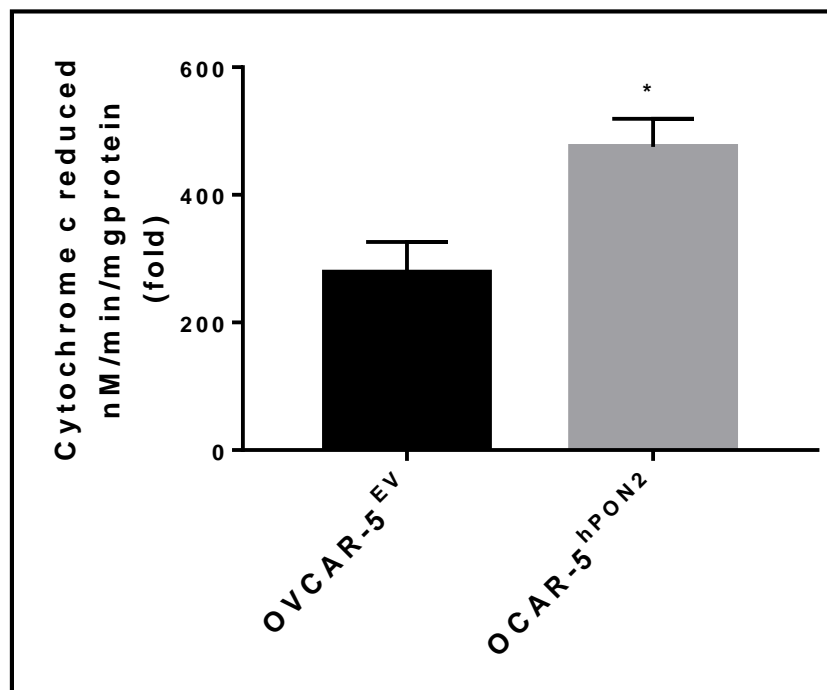

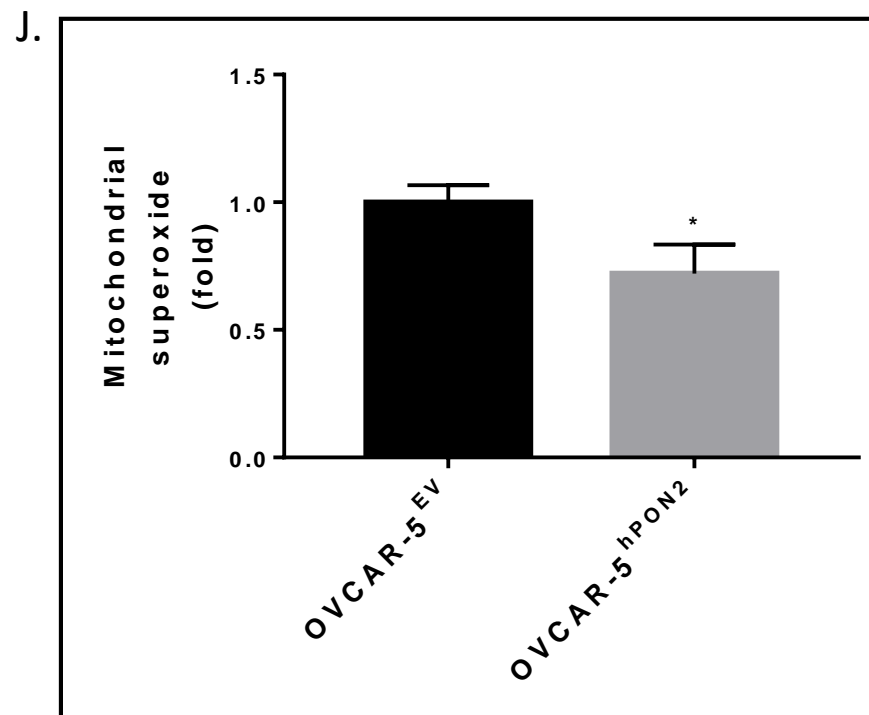

Supplement: Supplementary file 2 — Supplementary Figures(PDF 239 kb) [file 41419_2018_395_MOESM2_ESM.pdf]
