# Supplementary material for: Paraoxonase 2 overexpression inhibits tumor development in a mouse model of ovarian cancer
Source: Cell Death Dis. 2018 Mar 12;9(3):392. doi: 10.1038/s41419-018-0395-2 (PMC5847560; doi:10.1038/s41419-018-0395-2)
Supplement: Supplementary file 3 — Supplementary Table 1(PDF 32 kb) [file 41419_2018_395_MOESM3_ESM.pdf]

Supplementary Table 1

Probe Set ID	EV.mas5-Signal	PON2.mas5-Signal	Representative Public ID	Gene Symbol	Fold
1456078_x_at	23.88922	184.7433	BB012080	Tubb2c	7.7
1457795_at	16.39552	90.95401	BE956501	---	5.5
1449880_s_at	52.30219	204.0862	NM_007541	Bglap /// Bglap-rs1 /// Bglap2	3.9
1436846_x_at	2364.111	9174.489	AV024540	Gm10608 /// Gm2423 /// Ywhaq	3.9
1419974_at	31.01301	107.5268	C76618	Scp2	3.5
1440228_at	28.55656	98.34509	BB477637	Ranbp6	3.4
1455275_at	38.15729	131.1134	AW046441	E530001K10Rik	3.4
1460011_at	31.99199	109.7728	AW049789	Cyp26b1	3.4
1438102_at	28.55115	94.75272	AV271714	Senp8	3.3
1442980_at	30.06182	98.65948	BI410529	---	3.3
1434043_a_at	23.81319	76.94671	AV286809	Repin1	3.2
1450713_at	25.43243	80.25581	NM_013884	Cspg5	3.2
1437959_at	37.84633	109.7013	BB277175	Zfp324	2.9
1435595_at	39.47105	113.4743	AV016374	1810011O10Rik	2.9
1454003_at	26.34942	73.86738	AK009650	Afg3l2	2.8
1457299_at	65.14563	181.7207	BM941191	Grm4	2.8
1447084_at	34.37072	95.75336	BB376906	Nfatc1	2.8
1453426_a_at	47.14687	129.4027	AK005937	Wdfy1	2.7
1432557_at	26.89445	73.78678	AK006699	1700045I11Rik	2.7
1429580_x_at	46.51812	127.0013	AK016341	Spata24	2.7
1427688_a_at	27.81949	75.10371	D28531	Ptprs	2.7
1426514_at	104.3875	281.7295	AK019474	Chst15	2.7
1443789_x_at	53.95057	144.5798	AV206319	Cox8c	2.7
1425065_at	34.9502	93.5235	AB067535	Oas2	2.7
1415922_s_at	148.424	394.8299	NM_010807	Marcksl1	2.7
1417643_at	30.40571	80.63997	NM_025290	Rsph1	2.7
1420273_x_at	46.24959	122.0385	AV340322	Samhd1	2.6
1439380_x_at	34.76625	91.30497	BB093563	Meg3	2.6

1457351_at	67.35505	176.6096	AI451461	Taf2	2.6
1421061_at	33.97462	88.69366	NM_008189	Guca1a	2.6
1450282_at	55.1941	142.8608	NM_010202	Fgf4	2.6
1455910_at	73.03156	188.99	BB474449	Klhdc3	2.6
1451260_at	65.93419	168.8393	BC020001	Aldh1b1	2.6
1453281_at	28.94753	73.9517	BB700084	Pik3cd	2.6
1440108_at	49.40712	126.107	BM964154	Foxp2	2.6
1420396_at	30.6835	78.18363	NM_018767	Cd160	2.5
1441938_x_at	29.13997	74.1488	BB071777	Cables1	2.5
1454845_x_at	39.27888	98.36378	AW049955	Mchr1	2.5
1452853_at	31.42115	78.52033	AI790284	Shpk	2.5
1439407_x_at	3306.204	8185.104	AV212626	Tagln2	2.5
1421589_at	35.06266	86.72976	NM_010659	Krt31	2.5
1459877_x_at	965.292	2327.928	BB144036	Rnf185	2.4
1456427_at	30.9442	74.45177	BE988990	Gp1bb	2.4
1438974_x_at	65.9893	158.1201	BB207105	Pitpnm1	2.4
1423812_s_at	44.51345	106.3154	BC024822	Vopp1	2.4
1422777_at	34.90968	82.91729	NM_011795	C1ql1	2.4
1419872_at	33.77619	79.66445	AI323359	Csf1r	2.4
1460231_at	46.24128	109.0629	NM_012057	Irf5	2.4
1436346_at	83.19999	196.1614	AV246882	Cd109	2.4
1457162_at	55.0419	129.187	BB008111	Ldlrap1	2.3
1458850_at	44.03182	102.7031	BB479785	---	2.3
1447719_at	35.34515	81.91278	AV014271	Apoh	2.3
1440180_x_at	31.15613	72.1906	AV258279	Zbtb3	2.3
1454013_at	33.1882	76.64083	AK007938	1810062O18Rik	2.3
1421286_a_at	42.33493	97.52279	NM_018731	Atp4a	2.3
1440204_at	40.36106	92.76264	AW494150	3110039M20Rik	2.3
1456162_x_at	95.48545	218.6376	BB493359	Add3	2.3
1430385_a_at	37.28867	85.37067	BB207162	Glb1l	2.3

1436791_at	159.9284	365.5465	BB067079	Wnt5a	2.3
1445709_at	37.53068	85.31873	AI323780	Mdm1	2.3
1451813_at	32.10611	72.96208	L11065	Oprk1	2.3
1429277_at	31.61472	71.65097	AV063773	---	2.3
1427259_at	84.42247	191.0788	BB611004	Trim24	2.3
1432474_a_at	44.60037	100.6827	AK007999	Krtcap3	2.3
1443787_x_at	68.18481	153.461	BB004809	Casp14	2.3
1442238_a_at	35.77641	80.32942	BB450549	Kif6	2.2
1428613_at	69.82136	156.2601	AW105779	Ldhd	2.2
1430352_at	164.767	366.4375	BG141912	Adamts9	2.2
1435053_s_at	33.21909	73.82412	BB499987	Plekhh1	2.2
1418599_at	32.62	72.33852	NM_007729	Col11a1	2.2
1430310_at	64.64653	143.3089	BB535262	Tspan11	2.2
1424927_at	37.75106	83.56869	BC025083	Glipr1	2.2
1455515_at	66.072	146.1399	BQ177117	1810041L15Rik	2.2
1430214_a_at	37.83752	83.65699	BB103211	2610020H08Rik	2.2
1458994_at	32.88663	72.4516	BM215803	---	2.2
1429410_at	187.829	412.9061	AI595744	Eny2	2.2
1438955_x_at	140.886	308.0313	AV209130	Ppif	2.2
1437795_at	37.00518	80.90371	BM237841	---	2.2
1426208_x_at	40.19026	87.86121	AF147785	Plagl1	2.2
1446965_at	82.7272	180.7055	BG067578	Arhgef12	2.2
1438650_x_at	321.6903	699.6868	AV330726	Gja1	2.2
1452234_s_at	53.87327	116.8847	BF457957	Tmem191c	2.2
1456799_at	33.36722	72.36714	BB540910	E130110O22Rik	2.2
1418267_at	32.39088	70.17393	NM_008243	Mst1	2.2
1454159_a_at	35.41351	76.46683	AK011784	Igfbp2	2.2
1437375_at	191.2034	411.9077	BB745175	Rfx3	2.2
1438123_at	52.89206	113.4029	BB762627	Gm10033	2.1
1443071_at	32.34376	69.13246	BB140322	AI839979	2.1

1437339_s_at	59.14327	126.2	BB241731	Pcsk5	2.1
1441673_at	37.7277	80.35431	C79829	C80120	2.1
1417633_at	144.7771	308.3097	NM_011435	Sod3	2.1
1454858_x_at	398.0228	843.9843	AV171622	Mettl7a1	2.1
1460686_at	38.65071	81.71104	BC006866	Cntd1 /// LOC100044557	2.1
1424396_a_at	64.60932	136.5736	AU040643	Asrgl1	2.1
1454195_at	36.01013	75.78674	AK017041	4933433G19Rik	2.1
1452961_at	34.3229	72.16519	AK004681	1200009O22Rik	2.1
1432628_at	70.7207	148.3775	AV315574	Cbx3	2.1
1445439_at	112.3247	235.6493	AI413696	Epb4.9	2.1
1454227_at	44.47194	93.27142	AK015389	Htatip2	2.1
1422988_at	33.71734	70.64356	NM_018822	Sgsh	2.1
1457057_at	35.36471	73.88459	AV046807	1700042G07Rik	2.1
1432236_a_at	50.39218	104.9143	AK021174	Suv39h1	2.1
1435572_at	33.42677	69.46938	AV232798	2310014L17Rik	2.1
1440996_at	34.75532	71.6698	AV237452	---	2.1
1430798_x_at	98.61632	203.228	AV306676	Mrpl15	2.1
1449641_at	35.15779	72.4415	AI508733	Adk	2.1
1419995_at	46.69418	96.12805	AU015124	D10Ertd641e	2.1
1441580_at	47.53547	97.85639	AW551517	---	2.1
1421836_at	47.87509	98.49775	NM_008635	Mtap7	2.1
1426024_a_at	78.60142	161.6854	AF187147	Dbn1	2.1
1437641_at	65.71152	135.1163	BE981473	Rprd2	2.1
1435219_x_at	41.39734	84.79096	BG095529	Becn1	2.0
1452793_at	38.88223	79.60931	AI509011	Dzip1	2.0
1424428_at	151.0187	308.9098	BG065288	Ino80e	2.0
1433657_at	45.91542	93.79871	BI111848	Fam78a	2.0
1460681_at	89.34964	182.2524	BC024320	Ceacam1	2.0
1460738_at	40.15174	81.88788	AK012581	Limd2	2.0
1426147_s_at	89.17828	181.6088	BC021770	Cldn10a	2.0

1423555_a_at	72.95602	148.1509	BB329808	Ifi44	2.0
1422276_at	37.10217	75.13981	NM_020621	P2ry4	2.0
1458465_at	41.97506	84.89839	AW986124	Pan3	2.0
1452295_at	33.32828	67.28795	AV291712	Pmepa1	2.0
1421254_a_at	34.07418	68.69994	NM_011892	Sgcg	2.0
1420266_at	59.01332	118.6693	BB291769	Gm10627	2.0
1447070_at	171.1589	344.0064	AU018978	Eif4h	2.0
1444188_at	42.67551	85.72394	AI551516	---	2.0
1447647_at	41.47499	83.25345	BB129109	Wnt7a	2.0
1422548_at	74.33568	148.8821	BM233084	Dhdds	2.0
1450411_at	62.1122	124.3408	AV376206	Fam122a	2.0
1454181_at	35.06724	69.70596	AK015837	Fam164b	2.0
1450421_at	85.34879	169.4702	M92420	Tgfa	2.0
1454502_at	37.5021	74.45518	AK013567	2900022B07Rik	2.0
1418582_at	79.34223	157.3875	NM_009824	Cbfa2t3	2.0
1442286_at	39.432	78.14061	BB560335	---	2.0
1438446_x_at	72.22147	143.0275	BB530223	Inpp5k	2.0
1442147_at	45.19772	89.47643	BE948264	---	2.0
1431531_at	55.453	109.684	AK006327	Tex13a	2.0
1434563_at	125.535	248.2574	BM207149	Rps6kc1	2.0
1455192_at	59.34694	117.3261	BB385366	Tmem198	2.0
1436092_at	46.24756	91.41757	BB336256	---	2.0
1420349_at	48.61712	96.00736	NM_008966	Ptgfr	2.0
1437120_at	109.5977	216.3778	BB320376	Snx30	2.0
1437512_x_at	40.74778	80.4333	AV151335	Ebna1bp2	2.0
1435195_at	51.18452	100.9804	BG076358	Vash1	2.0
1438532_at	71.01306	139.8841	BB024475	Hmcn1	2.0
1452913_at	146.7773	288.629	AV337888	Pcp4l1	2.0
1436761_s_at	52.95817	104.1179	BB461323	Fam13c	2.0
1448818_at	52.87675	103.753	BC018425	Wnt5a	2.0

1421600_a_at	47.57869	93.27438	NM_030698	Trim26	2.0
1453614_a_at	45.83823	89.7327	AK017278	Nfe2l3	2.0
1428462_at	39.00462	76.11304	BB535888	Ppp2r5e	2.0
1422237_at	40.70679	79.33186	NM_008561	Mc3r	1.9
1433221_at	45.07095	87.77729	AK012008	2610311E24Rik	1.9
1446899_at	34.57824	67.30556	BB165801	---	1.9
1433358_at	37.19169	72.38439	AK020707	A230102O21Rik	1.9
1437892_at	99.19024	192.966	BQ084812	Zkscan3	1.9
1438797_at	60.95409	118.504	BB078988	---	1.9
1437509_x_at	43.10678	83.36929	BG095528	Prkd2	1.9
1429972_s_at	64.937	125.4666	BE948556	Txnrd2	1.9
1460265_at	68.44669	132.0774	AA672939	Pa2g4	1.9
1426454_at	136.5764	263.4302	AK002516	Arhgdib	1.9
1455691_at	61.41332	118.3463	BB819703	Cyp21a1	1.9
1453755_at	143.2225	275.6913	BM951302	Lsm11	1.9
1429836_at	36.03623	69.34348	BM218963	Uggt2	1.9
1429372_at	42.61528	81.97795	BM508495	Sox11	1.9
1444437_at	61.31558	117.8861	BB086152	Usp34	1.9
1447219_at	61.84616	118.8862	BQ031470	---	1.9
1423390_at	382.2245	734.5611	AV008871	Siah1a	1.9
1425914_a_at	69.95954	134.1277	BC021410	Armcx1	1.9
1417065_at	163.9695	313.4886	NM_007913	Egr1	1.9
1455569_at	47.42611	90.66695	BM239992	---	1.9
1421416_at	41.44918	79.20214	NM_011944	Map2k7	1.9
1431535_at	48.82946	93.22128	AK019494	Plb1	1.9
1450876_at	149.1534	284.7062	AI987976	Cfh /// LOC100048018	1.9
1434621_at	46.08301	87.82755	AV298104	Tmem204	1.9
1451048_at	36.58829	69.71835	BG086961	Metap2	1.9
1418541_at	43.29849	82.33905	NM_134046	Cenpo	1.9
1455367_at	45.33296	86.17818	BF020021	Dnd1	1.9

1457688_at	53.89444	102.421	AV343776	Zfp398	1.9
1419682_a_at	95.35551	181.1971	BB400773	2810408M09Rik /// Trp53rk	1.9
1460307_at	52.19938	99.14686	AF124142	Akt3	1.9
1426180_a_at	57.15529	108.5188	U82380	Smr2	1.9
1433505_a_at	464.2647	878.018	BB315861	Lrrc8d	1.9
1453304_s_at	327.307	618.765	BM245572	Ly6e	1.9
1430681_at	52.28293	98.72349	AI482548	Cryl1	1.9
1425994_a_at	73.35981	138.5046	AB037111	Asah2	1.9
1445203_at	50.94937	95.96177	AW122752	Pdzd2	1.9
1426960_a_at	36.31166	68.28625	BM118638	Fa2h	1.9
1435193_at	44.85991	84.31223	BB085904	A230050P20Rik	1.9
1423294_at	44.74443	84.08958	AW555393	Mest	1.9
1446325_at	66.00076	123.8899	AV325420	Pcyox1	1.9
1439153_at	37.68124	70.59167	AV274826	Rnf144b	1.9
1456857_at	168.2414	314.9083	BB782615	1500011B03Rik	1.9
1435033_at	40.97055	76.57968	AV024939	Arhgef4	1.9
1446521_at	38.91133	72.72345	BB538708	---	1.9
1420675_at	51.04276	95.31711	NM_019747	Zfp113	1.9
1444018_at	71.16959	132.8961	BB794854	B930098A02Rik	1.9
1416855_at	1222.832	2277.857	BB550400	Gas1	1.9
1422167_at	50.36301	93.80804	NM_009154	Sema5a	1.9
1440955_at	73.7794	137.3558	BQ126564	Kcp	1.9
1436954_at	43.72895	81.21959	C76969	Wipf1	1.9
1448690_at	104.9401	194.8109	NM_008430	Kcnk1	1.9
1417522_at	69.59713	129.1809	AF441120	Fbxo32	1.9
1426429_at	50.2167	93.1529	BM210680	Thap7	1.9
1447694_x_at	168.5955	312.5639	BB350308	Neo1	1.9
1446516_at	59.92137	111.0895	BB176719	Bcl7c	1.9
1457598_at	36.20046	67.07882	BE979944	Glrx3	1.9
1439261_x_at	39.35756	72.91457	AV102700	Mitd1	1.9

1440989_at	74.54712	138.0156	BB183373	Mrpl35	1.9
1456226_x_at	2449.2	4528.044	BB234940	Ddr1	1.8
1455607_at	36.94211	68.20489	BG072958	Rspo3	1.8
1437740_at	99.95065	184.4323	BB757269	Plekhm2	1.8
1440978_at	75.06935	138.3933	BE992189	Pigm	1.8
1450183_a_at	51.02778	94.02875	NM_008507	LOC100047863 /// Sh2b3	1.8
1447228_at	112.7856	207.6803	BM248411	Arfgap2	1.8
1418728_at	109.5552	201.6954	L36062	Star	1.8
1440860_at	36.63745	67.44804	BB126987	---	1.8
1450749_a_at	70.05975	128.9532	NM_013613	Nr4a2	1.8
1448990_a_at	200.9962	369.8426	AI255256	Myo1b	1.8
1447816_x_at	181.2305	333.3277	BB059395	Oxnad1	1.8
1424477_at	78.58682	144.4471	BC019731	Tmem184a	1.8
1441990_at	47.23685	86.79686	BM236098	Mre11a	1.8
1451417_at	96.6226	177.4525	U31625	Brca1	1.8
1451047_at	134.2903	246.3793	BI966443	Itm2a	1.8
1448945_at	84.9452	155.8343	BC024534	Pllp	1.8
1422789_at	149.5852	273.9095	NM_009022	Aldh1a2	1.8
1417155_at	121.2903	222.0607	BC005453	Mycn	1.8
1457534_at	38.09829	69.69342	BB481074	---	1.8
1459626_at	63.08202	115.3935	AW108268	---	1.8
1455167_at	198.2509	362.6489	AA144594	Cox8c	1.8
1455779_at	37.86696	69.24454	BQ175021	Mtap1a	1.8
1425248_a_at	74.48457	136.1437	AB000828	LOC100048488 /// Tyro3	1.8
1441902_x_at	147.6504	269.6649	BB264298	LOC100048058 /// Slc29a4	1.8
1427149_at	59.35387	108.1999	BB486127	Plekha6	1.8
1420570_x_at	511.3059	929.9877	NM_013772	Tcl1b3	1.8
1427513_at	45.92512	83.49495	BI144810	BC024137	1.8
1426405_at	62.41694	113.3171	BI150320	Rnf11	1.8
1436225_at	63.82357	115.8125	BB082386	Trpm2	1.8

1447491_at	49.06474	89.00909	BE987722	---	1.8
1455932_at	64.25619	116.4832	BE989081	Mtdh	1.8
1418268_at	42.01677	76.1431	NM_013561	Htr3a	1.8
1437432_a_at	54.00589	97.78355	BM244351	Trim12	1.8
1429686_at	148.7566	269.3264	BG070811	Polr3f	1.8
1418517_at	122.2289	221.222	NM_008393	Irx3	1.8
1450101_a_at	51.74082	93.58757	BB329638	Magi3	1.8
1417932_at	99.19846	179.3522	NM_008360	Il18	1.8
1443484_at	37.25906	67.26333	AW536522	Rpap1	1.8
1458302_at	67.73479	122.2273	BE200453	---	1.8
1439124_at	81.32647	146.566	BB747838	Wdr91	1.8
1459947_at	35.90819	64.68774	BB007036	---	1.8
1416200_at	73.53899	132.3364	NM_133775	Il33	1.8
1426073_at	54.29508	97.67815	AF292033	Twsg1	1.8
1442441_at	48.75018	87.6807	AV008016	Rnasek	1.8
1436069_at	908.9357	1634.366	AW545952	Ing5	1.8
1435986_x_at	991.4673	1776.597	AW107712	Sdhc	1.8
1438295_at	43.55664	78.03167	BM247146	---	1.8
1444387_at	79.47211	142.3631	BB409982	Nmt2	1.8
1450839_at	65.7998	117.8397	D45203	D0H4S114	1.8
1436756_x_at	84.0062	150.4138	BB114220	Hadh	1.8
1457121_at	71.12092	127.2657	AV271877	Obsl1	1.8
1420023_at	344.0756	615.3493	C77379	Etf1	1.8
1452388_at	148.9042	265.8887	AW763765	Hspa1a	1.8
1430025_at	100.4011	179.1674	AK015002	Ppp3cc	1.8
1423246_at	199.2256	355.4659	BI100077	Erp44	1.8
1425120_x_at	61.97311	110.5595	BC021795	Ifi27l2b	1.8
1445514_at	47.06657	83.83067	BE456323	---	1.8
1443521_at	47.75426	85.00492	BM218716	---	1.8
1436199_at	63.57329	113.142	AU042532	Trim14	1.8

1439877_at	51.69342	91.90749	BB125494	---	1.8
1449005_at	49.03044	87.16994	NM_030696	Slc16a3	1.8
1433556_at	56.61339	100.5379	AV264037	Adap1	1.8
1419937_at	78.60103	139.582	C76423	Gm12264	1.8
1442014_at	39.35235	69.84586	BB531645	---	1.8
1442618_at	44.06115	78.19694	BB477709	---	1.8
1460116_s_at	540.4299	959.0165	AI450584	Spred1	1.8
1451644_a_at	44.2395	78.48956	BC010602	H2-gs10 /// H2-Q6 /// LOC68395	1.8
1419013_at	210.9686	374.0206	NM_026181	Gpatch1	1.8
1433377_at	51.13276	90.64523	AK017990	5830445D09Rik	1.8
1448337_at	218.6967	386.9911	NM_018888	Uqcc	1.8
1440082_at	40.97391	72.48559	AW552837	Ptk2	1.8
1424722_at	45.52433	80.4093	BC021390	1300017J02Rik	1.8
1424492_at	60.84988	107.315	BC003841	Trpc2	1.8
1453138_at	44.96673	79.24553	AA560280	Rpusd2	1.8
1451701_x_at	72.61094	127.8357	BC012650	Cldn3	1.8
1422898_s_at	37.31843	65.6848	NM_009203	Slc22a12	1.8
1437189_x_at	51.19179	90.06923	BB311104	Eif3m	1.8
1437590_at	52.65476	92.53784	BB781167	Pyroxd2	1.8
1437422_at	333.9025	586.0797	AV375653	Sema5a	1.8
1426320_at	62.63898	109.9383	AF399755	Snx29	1.8
1456510_x_at	111.016	194.843	BB703414	Higd1c /// Mettl7a2	1.8
1453490_at	51.21278	89.88087	AK013338	Sass6	1.8
1458517_at	53.8586	94.51682	BI076610	---	1.8
1434150_a_at	413.3955	724.9001	AV171622	Higd1c /// Mettl7a1 /// Mettl7a2	1.8
1424020_at	1524.391	2671.573	BB837198	Arl6ip6	1.8
1448555_at	683.4571	1197.253	NM_028003	Rpap3	1.8
1420744_at	63.40411	111.0491	NM_009602	Chrnb2	1.8
1459679_s_at	304.9732	534.0571	AA406997	Myo1b	1.8
1455289_at	150.4193	263.3398	BQ174304	Ankrd13b	1.8

1442311_at	53.84117	94.23499	BM199880	Zfr	1.8
1436211_at	168.8924	96.41656	BB025874	Thoc4	-1.8
1460699_at	382.0823	218.0784	AA208652	Rps27	-1.8
1419523_at	944.6889	538.5001	NM_007819	Cyp3a13	-1.8
1425551_at	102.1392	58.2115	AA590970	Hip1r	-1.8
1418075_at	69.62106	39.62472	AK007601	St6galnac4	-1.8
1438764_at	172.9879	98.45342	AV333182	Anxa7	-1.8
1443870_at	4026.422	2291.16	BB291885	Abcc4	-1.8
1427691_a_at	205.3872	116.8538	Y09864	Ifnar2	-1.8
1452456_at	84.66731	48.14314	BE653335	Nrip2	-1.8
1429332_at	64.76343	36.80009	AK019505	4632427E13Rik	-1.8
1434350_at	196.572	111.6408	BG070296	Csrnp1	-1.8
1456904_at	270.3176	153.058	BQ101405	---	-1.8
1457019_s_at	89.17324	50.45937	BB241474	Rdh14	-1.8
1448965_at	119.6059	67.5839	BM238259	Ino80	-1.8
1440329_s_at	115.5164	65.22316	AV274050	Gpatch2	-1.8
1436469_at	78.09673	44.07708	BB022341	Brd7 /// LOC634327	-1.8
1460569_x_at	115.8578	65.29727	AW611462	Cldn3	-1.8
1452816_at	124.4892	70.14005	BB667813	Mlf1ip	-1.8
1431820_at	115.5743	65.03996	AK014549	4632404H12Rik	-1.8
1455937_at	78.16463	43.93401	BB085030	Lrrtm4	-1.8
1459920_at	137.4093	77.21995	AA163908	---	-1.8
1453427_at	85.78469	48.15017	AK011501	Csnk2a1	-1.8
1418246_at	67.24397	37.65074	BG277926	Rbm9	-1.8
1426834_s_at	92.01097	51.515	BM234701	D930015E06Rik	-1.8
1453419_at	368.3675	205.996	AK013800	Mras	-1.8
1444021_at	149.6666	83.43938	AW741388	---	-1.8
1438087_at	99.99837	55.73261	BB744467	Tpmt	-1.8
1444789_at	69.93337	38.9128	AI427762	---	-1.8
1421228_at	96.93822	53.93328	AF128193	Ccl7	-1.8

1455568_at	85.00188	47.2317	BI689456	Ccdc74a	-1.8
1458410_at	65.88718	36.46943	BB236521	Ralgapa1	-1.8
1459879_at	66.07206	36.47944	AW492820	4921513D23Rik	-1.8
1422907_at	64.85963	35.78188	NM_008141	Gnat2	-1.8
1420080_a_at	77.80882	42.86752	AI324119	Gfpt1	-1.8
1441305_at	66.1043	36.36211	BB360028	Nedd4l	-1.8
1425055_at	181.7741	99.56871	BB532544	Saps2	-1.8
1424217_at	101.9754	55.73724	U52197	Papola	-1.8
1442347_at	415.9109	227.038	AI848122	Lrp8	-1.8
1426560_a_at	77.31334	42.14279	AA223007	Npnt	-1.8
1438900_at	70.76479	38.54501	BB141248	Sacm1l	-1.8
1439150_x_at	75.93301	41.25379	BB480256	Grtp1	-1.8
1447935_at	101.7785	55.1409	BB499286	Fancm	-1.8
1430302_at	69.25632	37.48213	AK019934	Cnrip1	-1.8
1445239_at	73.45669	39.54973	BG066348	Gatad2a	-1.9
1426383_at	93.88725	50.53202	BF303057	Cry2	-1.9
1457465_at	133.3966	71.74062	BB496399	Shroom4	-1.9
1422575_at	91.69454	49.28726	BE291523	Mxd4	-1.9
1456963_at	90.6759	48.72779	BM508503	Tmem214	-1.9
1422494_s_at	216.4537	116.2628	NM_053180	Cdk20	-1.9
1447426_at	79.13467	42.44849	BF465664	---	-1.9
1416275_at	136.8665	73.00966	NM_134420	Slc26a6	-1.9
1435516_x_at	86.61299	46.19481	BB752007	Rtel1	-1.9
1419808_at	78.15502	41.63964	AU022131	Cog4	-1.9
1460390_at	81.31628	43.31092	BI648081	Sorl1	-1.9
1418486_at	5872.52	3125.078	NM_011704	Vnn1	-1.9
1452409_at	117.5294	62.54122	BB105833	Gltscr2	-1.9
1447869_x_at	100.6797	53.49622	AV047988	Rhobtb3	-1.9
1442072_at	99.31362	52.68018	BM227237	C230081A13Rik	-1.9
1420284_at	338.6364	179.1432	BB087707	Bat4	-1.9

1458634_at	69.83517	36.89606	BB254163	---	-1.9
1451718_at	67.70773	35.68048	BB768495	Plp1	-1.9
1429639_at	77.37474	40.74513	AK009137	Gpcpd1	-1.9
1424714_at	141.765	74.55026	BC008184	Aldoc	-1.9
1458493_a_at	71.23511	37.44555	BE982367	2410089E03Rik	-1.9
1447739_x_at	173.9447	91.10249	AV294746	Klhdc4	-1.9
1423290_at	463.8855	242.8137	BM231738	Hyou1	-1.9
1430158_at	99.21716	51.78059	AV019837	3110021A11Rik	-1.9
1425636_at	93.77979	48.81583	BC008159	Hhat	-1.9
1445827_at	74.37977	38.70609	BB223862	Zmynd8	-1.9
1458130_at	101.496	52.75873	BB539454	---	-1.9
1427379_at	68.02306	35.15222	BC020092	Pnpla6	-1.9
1445147_at	66.02085	34.06304	BB226416	Psme4	-1.9
1457593_at	73.52772	37.93322	BM221021	2610202C22Rik	-1.9
1456143_at	135.8534	69.65861	BM203042	Zmynd8	-2.0
1420533_at	85.7509	43.89705	AK004815	Gucy1a3	-2.0
1427588_a_at	213.4624	109.0616	X95226	Dtna	-2.0
1425075_at	202.5458	103.3995	AF411837	Gatad2b	-2.0
1457268_at	293.6314	149.7837	BM199751	Dot1l	-2.0
1450131_a_at	74.09614	37.71557	NM_138653	Bspry	-2.0
1430633_s_at	152.3568	77.54066	BG066234	C430045I18Rik	-2.0
1435402_at	162.541	82.64716	BB066186	Gramd1b	-2.0
1427762_x_at	146.2983	74.27309	M25487	Hist1h2bp	-2.0
1430752_at	80.84177	40.8042	BB397948	C330006D17Rik	-2.0
1458330_x_at	328.4265	165.4396	BB236001	Cnnm3	-2.0
1417824_at	74.12189	37.23238	AK013138	Gcat	-2.0
1448695_at	165.0978	82.67136	NM_008857	Prkci	-2.0
1422709_a_at	306.7921	152.3328	NM_020603	Wdr46	-2.0
1424756_at	71.77329	35.42778	BB320674	Hip1	-2.0
1457356_at	120.2021	59.27718	BI793602	---	-2.0

1433479_at	143.5557	70.74186	AV030071	Ubr7	-2.0
1453976_at	69.63848	34.27633	BB624514	4432414F05Rik	-2.0
1421524_at	70.32849	34.41537	NM_007685	Cfc1	-2.0
1446417_at	152.4911	74.57935	AW553625	---	-2.0
1432391_at	138.5949	67.58305	AK010621	Ccdc21	-2.1
1446761_at	67.50484	32.87302	C77659	D8Ertd56e	-2.1
1456154_at	74.10059	36.04917	BE981638	---	-2.1
1448154_at	229.2285	111.2306	NM_013864	Ndrg2	-2.1
1418008_at	226.8635	109.94	BM932567	Gcfc1	-2.1
1452244_at	543.998	261.2499	AK018128	6330406I15Rik	-2.1
1429949_at	176.1349	84.2381	AK018340	6530415H11Rik	-2.1
1447845_s_at	2615.792	1248.393	AV360029	Vnn1	-2.1
1421058_at	236.94	112.9994	NM_009626	Adh7	-2.1
1439386_x_at	162.7156	77.56166	BB319038	Mat2a	-2.1
1437318_at	127.4179	60.65614	BB468082	Pak3	-2.1
1460419_a_at	195.1289	92.80601	X59274	Prkcb	-2.1
1429272_a_at	120.1201	56.68604	AK018646	Apol7a /// Apol7c /// Gm8221	-2.1
1421407_at	84.72254	39.86009	NM_010170	F2rl2	-2.1
1418873_at	115.8717	54.49319	NM_053198	Sfxn4	-2.1
1444451_at	92.56303	43.48133	BB101283	Pappa2	-2.1
1440982_at	79.89532	37.33963	BB209400	LOC552909	-2.1
1420661_a_at	115.6781	53.97613	NM_025757	4933439F18Rik	-2.1
1416740_at	84.97235	39.50938	AW744319	Col5a1	-2.2
1420796_at	329.3627	153.0769	NM_009644	Ahrr	-2.2
1428476_a_at	276.1171	128.3245	AK003759	Elac2	-2.2
1441988_at	197.7707	90.91176	AI482429	Ppm1k	-2.2
1441124_at	87.05923	39.90593	AV224430	Vezt	-2.2
1457231_at	74.05178	33.93	BB409314	---	-2.2
1453497_a_at	69.23957	31.35507	AK014488	Piga	-2.2
1452014_a_at	130.0407	57.67058	AF440694	Igf1	-2.3

1424490_at	225.5156	99.66274	BC024802	Zfp428	-2.3
1434091_at	95.98101	42.20129	BB189927	Faah	-2.3
1459829_x_at	71.34475	31.22421	BB380616	BC031781	-2.3
1445199_at	80.35799	34.389	BM942479	---	-2.3
1458518_at	83.76444	35.40852	BM935843	Cpeb2	-2.4
1454780_at	78.88351	33.34007	AV238718	Galntl4	-2.4
1448485_at	302.2766	127.2738	NM_008116	Ggt1	-2.4
1457367_at	79.63764	33.28411	BB667309	---	-2.4
1426127_x_at	311.258	129.2054	AF288377	Klra18	-2.4
1455803_at	957.4942	396.7173	AV375182	Slco4a1	-2.4
1439722_at	72.27216	29.79593	BB165972	---	-2.4
1424555_at	135.802	55.92248	BC004010	9430015G10Rik	-2.4
1447551_x_at	113.9197	46.55214	BB274232	Lphn3	-2.4
1454844_at	116.1021	47.25822	AW049955	Mchr1	-2.5
1450475_at	73.47749	29.85147	U79738	Dlx3	-2.5
1436610_at	83.27338	33.79387	BQ176856	Ankrd12	-2.5
1441792_at	79.98077	32.43979	BB788678	A630033E08Rik	-2.5
1457528_at	209.8298	84.81692	BB454531	Slc4a7	-2.5
1440781_at	85.33616	34.12018	BB333095	B830007D08Rik	-2.5
1460373_a_at	88.69019	35.10062	AY037804	Setd4	-2.5
1451594_s_at	99.30023	39.19837	AF425084	Serpinb6c	-2.5
1453413_at	87.8941	34.6764	AW048640	Gnas	-2.5
1458425_at	94.75455	36.692	BM945176	---	-2.6
1419127_at	92.73621	35.81265	NM_023456	Npy	-2.6
1460054_at	73.68597	28.3902	AW491655	Trappc6a	-2.6
1446278_at	100.856	38.1943	BB077029	---	-2.6
1418416_x_at	112.396	42.25665	NM_020261	Psg23	-2.7
1429683_at	207.367	76.97845	BG094398	Ttpal	-2.7
1427072_at	302.7924	111.8802	BM119481	Stard8	-2.7
1441326_at	144.9783	52.56334	AV381752	Cp	-2.8

1421060_at	79.18677	28.22737	AF312858	Mllt1	-2.8
1447802_x_at	169.1138	60.17278	AV099323	---	-2.8
1418657_at	112.6268	39.55287	BC019383	Ino80b	-2.8
1437114_at	103.4454	35.23602	AV230892	A930001N09Rik	-2.9
1438793_x_at	127.9656	39.36474	BB222127	Ergic1	-3.3
1448929_at	658.825	160.5595	NM_028784	F13a1	-4.1
1452944_at	157.7719	37.35467	AK004030	Afmid	-4.2
1449804_at	137.8646	30.54066	AV380429	Pnmt	-4.5
1447484_x_at	136.4588	29.62913	AV011566	Snhg7	-4.6
1422217_a_at	592.4177	113.0746	NM_009992	Cyp1a1	-5.2
