# Supplementary material for: Paraoxonase 2 overexpression inhibits tumor development in a mouse model of ovarian cancer
Source: Cell Death Dis. 2018 Mar 12;9(3):392. doi: 10.1038/s41419-018-0395-2 (PMC5847560; doi:10.1038/s41419-018-0395-2)
Supplement: Supplementary file 4 — Supplementary Table 2(PDF 82 kb) [file 41419_2018_395_MOESM4_ESM.pdf]

Supplementary Table 2	
**Ingenuity Canonical Pathways**	**-log(p-value)**
Adipogenesis pathway	-0E00
BMP signaling pathway	-0E00
Caveolar-mediated Endocytosis Signaling	-0E00
Cellular Effects of Sildenafil (Viagra)	-0E00
GPCR-Mediated Integration of Enteroendocrine Signaling Exemplified by an L Cell	-0E00
Glutamate Receptor Signaling	-0E00
Gustation Pathway	-0E00
Hypoxia Signaling in the Cardiovascular System	-0E00
Induction of Apoptosis by HIV1	-0E00
Mitochondrial Dysfunction	-0E00
Mitotic Roles of Polo-Like Kinase	-0E00
Oxidative Phosphorylation	-0E00
Pyridoxal 5'-phosphate Salvage Pathway	-0E00
Remodeling of Epithelial Adherens Junctions	-0E00
Salvage Pathways of Pyrimidine Ribonucleotides	-0E00
T Helper Cell Differentiation	-0E00
p38 MAPK Signaling	-0E00
phagosome maturation	-0E00
Dermatan Sulfate Biosynthesis	1.99E-01
Heparan Sulfate Biosynthesis	1.99E-01
Phospholipases	1.99E-01
OX40 Signaling Pathway	2.04E-01
Role of IL-17A in Arthritis	2.04E-01
Chondroitin Sulfate Biosynthesis	2.14E-01
CD27 Signaling in Lymphocytes	2.19E-01
Assembly of RNA Polymerase II Complex	2.24E-01
Allograft Rejection Signaling	2.35E-01
Heparan Sulfate Biosynthesis (Late Stages)	2.35E-01
TNFR1 Signaling	2.41E-01
Type I Diabetes Mellitus Signaling	2.44E-01
Chondroitin Sulfate Biosynthesis (Late Stages)	2.59E-01
Autoimmune Thyroid Disease Signaling	2.73E-01
Dermatan Sulfate Biosynthesis (Late Stages)	2.73E-01
Role of IL-17F in Allergic Inflammatory Airway Diseases	2.8E-01
Neuroprotective Role of THOP1 in Alzheimer's Disease	2.87E-01
Transcriptional Regulatory Network in Embryonic Stem Cells	2.87E-01
Antioxidant Action of Vitamin C	2.91E-01
Thyroid Cancer Signaling	2.95E-01
Antigen Presentation Pathway	3.11E-01
Death Receptor Signaling	3.11E-01
Complement System	3.19E-01
Crosstalk between Dendritic Cells and Natural Killer Cells	3.21E-01

Factors Promoting Cardiogenesis in Vertebrates	3.21E-01
Epithelial Adherens Junction Signaling	3.22E-01
Apoptosis Signaling	3.27E-01
Cell Cycle Regulation by BTG Family Proteins	3.28E-01
Coagulation System	3.28E-01
Regulation of Actin-based Motility by Rho	3.32E-01
TGF-β Signaling	3.32E-01
UVA-Induced MAPK Signaling	3.32E-01
IL-9 Signaling	3.37E-01
Interferon Signaling	3.37E-01
Oncostatin M Signaling	3.37E-01
D-myo-inositol-5-phosphate Metabolism	3.44E-01
Circadian Rhythm Signaling	3.46E-01
Role of JAK2 in Hormone-like Cytokine Signaling	3.56E-01
Cytotoxic T Lymphocyte-mediated Apoptosis of Target Cells	3.66E-01
Superpathway of Methionine Degradation	3.66E-01
Altered T Cell and B Cell Signaling in Rheumatoid Arthritis	3.67E-01
Cardiac β-adrenergic Signaling	3.72E-01
ILK Signaling	3.87E-01
Role of p14/p19ARF in Tumor Suppression	3.88E-01
Dopamine Receptor Signaling	3.93E-01
Estrogen Receptor Signaling	3.98E-01
Intrinsic Prothrombin Activation Pathway	4E-01
Agranulocyte Adhesion and Diapedesis	4.14E-01
STAT3 Pathway	4.22E-01
Antiproliferative Role of TOB in T Cell Signaling	4.25E-01
D-myo-inositol (1,4,5,6)-Tetrakisphosphate Biosynthesis	4.25E-01
D-myo-inositol (3,4,5,6)-tetrakisphosphate Biosynthesis	4.25E-01
EIF2 Signaling	4.28E-01
Toll-like Receptor Signaling	4.29E-01
RhoA Signaling	4.36E-01
Calcium Signaling	4.37E-01
Gluconeogenesis I	4.38E-01
Glycolysis I	4.52E-01
IL-22 Signaling	4.52E-01
Role of JAK1, JAK2 and TYK2 in Interferon Signaling	4.52E-01
Superpathway of D-myo-inositol (1,4,5)-trisphosphate Metabolism	4.52E-01
Triacylglycerol Degradation	4.52E-01
Basal Cell Carcinoma Signaling	4.52E-01
TREM1 Signaling	4.52E-01
cAMP-mediated signaling	4.59E-01
GDNF Family Ligand-Receptor Interactions	4.61E-01
IL-10 Signaling	4.61E-01
Granulocyte Adhesion and Diapedesis	4.62E-01

PPARα/RXRα Activation	4.62E-01
TCA Cycle II (Eukaryotic)	4.67E-01
GABA Receptor Signaling	4.77E-01
Polyamine Regulation in Colon Cancer	4.83E-01
CD40 Signaling	4.95E-01
IL-17A Signaling in Airway Cells	4.95E-01
Cell Cycle: G1/S Checkpoint Regulation	5.04E-01
ERK5 Signaling	5.04E-01
PXR/RXR Activation	5.04E-01
Protein Ubiquitination Pathway	5.13E-01
Role of PI3K/AKT Signaling in the Pathogenesis of Influenza	5.23E-01
NGF Signaling	5.27E-01
Role of JAK1 and JAK3 in γc Cytokine Signaling	5.33E-01
1D-myo-inositol Hexakisphosphate Biosynthesis II (Mammalian)	5.35E-01
D-myo-inositol (1,3,4)-trisphosphate Biosynthesis	5.35E-01
GADD45 Signaling	5.35E-01
Aldosterone Signaling in Epithelial Cells	5.4E-01
ATM Signaling	5.43E-01
3-phosphoinositide Biosynthesis	5.53E-01
Cysteine Biosynthesis III (mammalia)	5.55E-01
D-myo-inositol (1,4,5)-trisphosphate Degradation	5.55E-01
phagosome formation	5.56E-01
HIF1α Signaling	5.72E-01
Actin Nucleation by ARP-WASP Complex	5.74E-01
ErbB2-ErbB3 Signaling	5.74E-01
Regulation of Cellular Mechanics by Calpain Protease	5.85E-01
Superpathway of Melatonin Degradation	5.85E-01
Wnt/Ca+ pathway	5.85E-01
Amyotrophic Lateral Sclerosis Signaling	5.96E-01
Lymphotoxin β Receptor Signaling	5.96E-01
Extrinsic Prothrombin Activation Pathway	5.99E-01
Methionine Degradation I (to Homocysteine)	5.99E-01
Unfolded protein response	6.08E-01
Superpathway of Inositol Phosphate Compounds	6.13E-01
3-phosphoinositide Degradation	6.19E-01
Role of Cytokines in Mediating Communication between Immune Cells	6.2E-01
Mouse Embryonic Stem Cell Pluripotency	6.21E-01
Leukotriene Biosynthesis	6.23E-01
Telomere Extension by Telomerase	6.23E-01
γ-glutamyl Cycle	6.23E-01
Fcγ Receptor-mediated Phagocytosis in Macrophages and Monocytes	6.3E-01
Nur77 Signaling in T Lymphocytes	6.32E-01
Hepatic Fibrosis / Hepatic Stellate Cell Activation	6.44E-01
Amyloid Processing	6.45E-01

Melatonin Degradation I	6.45E-01
Aryl Hydrocarbon Receptor Signaling	6.47E-01
DNA Double-Strand Break Repair by Non-Homologous End Joining	6.49E-01
Vitamin-C Transport	6.49E-01
PPAR Signaling	6.57E-01
CNTF Signaling	6.58E-01
Cell Cycle: G2/M DNA Damage Checkpoint Regulation	6.58E-01
Ephrin A Signaling	6.71E-01
Bile Acid Biosynthesis, Neutral Pathway	6.78E-01
NAD biosynthesis II (from tryptophan)	6.78E-01
Cdc42 Signaling	6.93E-01
MSP-RON Signaling Pathway	6.99E-01
FGF Signaling	7.06E-01
HIPPO signaling	7.06E-01
TR/RXR Activation	7.06E-01
Cleavage and Polyadenylation of Pre-mRNA	7.09E-01
Glutaryl-CoA Degradation	7.09E-01
Role of Oct4 in Mammalian Embryonic Stem Cell Pluripotency	7.13E-01
Melanocyte Development and Pigmentation Signaling	7.16E-01
GPCR-Mediated Nutrient Sensing in Enteroendocrine Cells	7.27E-01
Communication between Innate and Adaptive Immune Cells	7.37E-01
Serotonin Receptor Signaling	7.43E-01
Atherosclerosis Signaling	7.6E-01
Gαi Signaling	7.69E-01
FcγRIIB Signaling in B Lymphocytes	7.75E-01
Mechanisms of Viral Exit from Host Cells	7.75E-01
Role of Hypercytokinemia/hyperchemokinemia in the Pathogenesis of Influenza	7.75E-01
B Cell Activating Factor Signaling	7.92E-01
Cyclins and Cell Cycle Regulation	7.93E-01
Hepatic Cholestasis	8.07E-01
Docosahexaenoic Acid (DHA) Signaling	8.09E-01
Netrin Signaling	8.09E-01
April Mediated Signaling	8.27E-01
CD28 Signaling in T Helper Cells	8.35E-01
PKCθ Signaling in T Lymphocytes	8.35E-01
Role of Wnt/GSK-3β Signaling in the Pathogenesis of Influenza	8.42E-01
Estrogen Biosynthesis	8.46E-01
IL-17 Signaling	8.55E-01
JAK/Stat Signaling	8.55E-01
Integrin Signaling	8.61E-01
autophagy	8.65E-01
FLT3 Signaling in Hematopoietic Progenitor Cells	8.68E-01
PEDF Signaling	8.68E-01
Glucocorticoid Biosynthesis	8.69E-01

Sphingosine and Sphingosine-1-phosphate Metabolism	8.69E-01
Sucrose Degradation V (Mammalian)	8.69E-01
Superoxide Radicals Degradation	8.69E-01
Protein Kinase A Signaling	8.8E-01
Corticotropin Releasing Hormone Signaling	8.96E-01
Role of Tissue Factor in Cancer	8.96E-01
Colorectal Cancer Metastasis Signaling	9.07E-01
Chemokine Signaling	9.08E-01
Melatonin Signaling	9.08E-01
Agrin Interactions at Neuromuscular Junction	9.22E-01
Neurotrophin/TRK Signaling	9.22E-01
Mineralocorticoid Biosynthesis	9.24E-01
Tryptophan Degradation to 2-amino-3-carboxymuconate Semialdehyde	9.24E-01
iCOS-iCOSL Signaling in T Helper Cells	9.4E-01
Regulation of eIF4 and p70S6K Signaling	9.46E-01
Inhibition of Angiogenesis by TSP1	9.49E-01
G Protein Signaling Mediated by Tubby	9.72E-01
Neuropathic Pain Signaling In Dorsal Horn Neurons	9.87E-01
Ceramide Degradation	9.87E-01
Thioredoxin Pathway	9.87E-01
UDP-N-acetyl-D-glucosamine Biosynthesis II	9.87E-01
Cardiac Hypertrophy Signaling	9.88E-01
Fatty Acid β-oxidation I	9.96E-01
Eicosanoid Signaling	9.98E-01
PCP pathway	9.98E-01
AMPK Signaling	9.98E-01
Paxillin Signaling	9.99E-01
p53 Signaling	9.99E-01
T Cell Receptor Signaling	1.02E00
Telomerase Signaling	1.02E00
Antiproliferative Role of Somatostatin Receptor 2	1.03E00
G-Protein Coupled Receptor Signaling	1.05E00
SAPK/JNK Signaling	1.06E00
Calcium-induced T Lymphocyte Apoptosis	1.06E00
Actin Cytoskeleton Signaling	1.07E00
Dendritic Cell Maturation	1.07E00
IL-15 Production	1.07E00
Glioma Invasiveness Signaling	1.08E00
IL-1 Signaling	1.09E00
Insulin Receptor Signaling	1.09E00
Tight Junction Signaling	1.1E00
Acetone Degradation I (to Methylglyoxal)	1.1E00
Role of CHK Proteins in Cell Cycle Checkpoint Control	1.12E00
FXR/RXR Activation	1.12E00

PAK Signaling	1.13E00
CTLA4 Signaling in Cytotoxic T Lymphocytes	1.14E00
Catecholamine Biosynthesis	1.15E00
Nicotine Degradation II	1.16E00
UVB-Induced MAPK Signaling	1.16E00
Bladder Cancer Signaling	1.16E00
Bupropion Degradation	1.16E00
LXR/RXR Activation	1.17E00
α-Adrenergic Signaling	1.17E00
Endometrial Cancer Signaling	1.18E00
Semaphorin Signaling in Neurons	1.18E00
Dopamine-DARPP32 Feedback in cAMP Signaling	1.19E00
Phototransduction Pathway	1.2E00
Role of Pattern Recognition Receptors in Recognition of Bacteria and Viruses	1.2E00
Germ Cell-Sertoli Cell Junction Signaling	1.2E00
HMGB1 Signaling	1.21E00
Leukocyte Extravasation Signaling	1.22E00
Synaptic Long Term Potentiation	1.23E00
Phospholipase C Signaling	1.24E00
Ceramide Signaling	1.25E00
Type II Diabetes Mellitus Signaling	1.26E00
Dopamine Degradation	1.27E00
D-glucuronate Degradation I	1.28E00
S-adenosyl-L-methionine Biosynthesis	1.28E00
Nicotine Degradation III	1.28E00
Regulation of IL-2 Expression in Activated and Anergic T Lymphocytes	1.28E00
Role of BRCA1 in DNA Damage Response	1.28E00
VDR/RXR Activation	1.3E00
nNOS Signaling in Neurons	1.31E00
Tryptophan Degradation III (Eukaryotic)	1.31E00
IL-8 Signaling	1.32E00
Glioblastoma Multiforme Signaling	1.33E00
Role of NANOG in Mammalian Embryonic Stem Cell Pluripotency	1.33E00
mTOR Signaling	1.33E00
Gαs Signaling	1.34E00
DNA damage-induced 14-3-3σ Signaling	1.35E00
Ethanol Degradation IV	1.35E00
Graft-versus-Host Disease Signaling	1.35E00
Production of Nitric Oxide and Reactive Oxygen Species in Macrophages	1.36E00
Pancreatic Adenocarcinoma Signaling	1.37E00
Ephrin B Signaling	1.37E00
LPS-stimulated MAPK Signaling	1.37E00
Leptin Signaling in Obesity	1.37E00
Prolactin Signaling	1.37E00

Melanoma Signaling	1.4E00
UVC-Induced MAPK Signaling	1.4E00
Sertoli Cell-Sertoli Cell Junction Signaling	1.43E00
Ephrin Receptor Signaling	1.44E00
RhoGDI Signaling	1.44E00
Anandamide Degradation	1.45E00
Asparagine Degradation I	1.45E00
Dolichol and Dolichyl Phosphate Biosynthesis	1.45E00
Growth Hormone Signaling	1.45E00
Systemic Lupus Erythematosus Signaling	1.47E00
Putrescine Degradation III	1.48E00
CCR5 Signaling in Macrophages	1.49E00
Role of MAPK Signaling in the Pathogenesis of Influenza	1.49E00
Relaxin Signaling	1.49E00
CDK5 Signaling	1.5E00
IL-15 Signaling	1.51E00
Role of NFAT in Regulation of the Immune Response	1.51E00
Wnt/β-catenin Signaling	1.51E00
Fatty Acid α-oxidation	1.54E00
Oxidative Ethanol Degradation III	1.54E00
Nitric Oxide Signaling in the Cardiovascular System	1.55E00
Estrogen-Dependent Breast Cancer Signaling	1.57E00
Glucocorticoid Receptor Signaling	1.59E00
DNA Double-Strand Break Repair by Homologous Recombination	1.59E00
Chronic Myeloid Leukemia Signaling	1.6E00
VEGF Signaling	1.65E00
Virus Entry via Endocytic Pathways	1.65E00
PI3K/AKT Signaling	1.65E00
ErbB4 Signaling	1.69E00
FAK Signaling	1.71E00
Neuregulin Signaling	1.71E00
RANK Signaling in Osteoclasts	1.71E00
Gα12/13 Signaling	1.72E00
Histamine Degradation	1.72E00
EGF Signaling	1.74E00
Adenine and Adenosine Salvage VI	1.75E00
Methylglyoxal Degradation VI	1.75E00
Threonine Degradation II	1.75E00
Serotonin Degradation	1.77E00
Thrombopoietin Signaling	1.77E00
CCR3 Signaling in Eosinophils	1.79E00
Retinoate Biosynthesis I	1.79E00
IL-2 Signaling	1.82E00
NRF2-mediated Oxidative Stress Response	1.83E00

Prostate Cancer Signaling	1.84E00
Androgen Signaling	1.84E00
Reelin Signaling in Neurons	1.86E00
Sphingosine-1-phosphate Signaling	1.87E00
Acute Myeloid Leukemia Signaling	1.88E00
LPS/IL-1 Mediated Inhibition of RXR Function	1.88E00
PDGF Signaling	1.9E00
HER-2 Signaling in Breast Cancer	1.92E00
VEGF Family Ligand-Receptor Interactions	1.92E00
CREB Signaling in Neurons	1.94E00
Acute Phase Response Signaling	1.95E00
eNOS Signaling	1.95E00
Rac Signaling	1.97E00
IL-4 Signaling	2E00
NF-κB Activation by Viruses	2E00
Cholecystokinin/Gastrin-mediated Signaling	2.01E00
Human Embryonic Stem Cell Pluripotency	2.02E00
IL-12 Signaling and Production in Macrophages	2.02E00
Small Cell Lung Cancer Signaling	2.05E00
Renal Cell Carcinoma Signaling	2.1E00
Huntington's Disease Signaling	2.1E00
Hereditary Breast Cancer Signaling	2.11E00
Breast Cancer Regulation by Stathmin1	2.12E00
Macropinocytosis Signaling	2.12E00
Tec Kinase Signaling	2.14E00
Erythropoietin Signaling	2.15E00
PI3K Signaling in B Lymphocytes	2.16E00
Angiopoietin Signaling	2.21E00
Clathrin-mediated Endocytosis Signaling	2.21E00
Role of Macrophages, Fibroblasts and Endothelial Cells in Rheumatoid Arthritis	2.22E00
Regulation of the Epithelial-Mesenchymal Transition Pathway	2.24E00
P2Y Purigenic Receptor Signaling Pathway	2.26E00
GM-CSF Signaling	2.29E00
G Beta Gamma Signaling	2.29E00
14-3-3-mediated Signaling	2.3E00
IL-6 Signaling	2.3E00
Gαq Signaling	2.32E00
Role of NFAT in Cardiac Hypertrophy	2.33E00
Sperm Motility	2.34E00
ErbB Signaling	2.37E00
Myc Mediated Apoptosis Signaling	2.42E00
Fc Epsilon RI Signaling	2.45E00
Endothelin-1 Signaling	2.47E00
Signaling by Rho Family GTPases	2.47E00

Natural Killer Cell Signaling	2.47E00
Renin-Angiotensin Signaling	2.47E00
Tryptophan Degradation X (Mammalian, via Tryptamine)	2.49E00
fMLP Signaling in Neutrophils	2.49E00
HGF Signaling	2.56E00
GNRH Signaling	2.68E00
ERK/MAPK Signaling	2.69E00
Ethanol Degradation II	2.71E00
IL-3 Signaling	2.76E00
PTEN Signaling	2.88E00
p70S6K Signaling	2.88E00
Non-Small Cell Lung Cancer Signaling	2.96E00
B Cell Receptor Signaling	2.97E00
NF-κB Signaling	3E00
Xenobiotic Metabolism Signaling	3.11E00
Thrombin Signaling	3.23E00
Ovarian Cancer Signaling	3.24E00
Role of Osteoblasts, Osteoclasts and Chondrocytes in Rheumatoid Arthritis	3.29E00
CXCR4 Signaling	3.39E00
Glioma Signaling	3.54E00
Molecular Mechanisms of Cancer	3.54E00
Noradrenaline and Adrenaline Degradation	3.61E00
Synaptic Long Term Depression	3.75E00
RAR Activation	3.82E00
Axonal Guidance Signaling	4.02E00
Gap Junction Signaling	4.04E00
IGF-1 Signaling	4.2E00
