# Supplementary material for: Paraoxonase 2 overexpression inhibits tumor development in a mouse model of ovarian cancer
Source: Cell Death Dis. 2018 Mar 12;9(3):392. doi: 10.1038/s41419-018-0395-2 (PMC5847560; doi:10.1038/s41419-018-0395-2)
Supplement: Supplementary file 5 — Supplementary Table 3(PDF 55 kb) [file 41419_2018_395_MOESM5_ESM.pdf]

Supplementary Table 3	
**Ingenuity Canonical Pathways**	**-log(p-value)**
TNFR1 Signaling	2.41E-01
Death Receptor Signaling	3.11E-01
TGF-β Signaling	3.32E-01
Role of p14/p19ARF in Tumor Suppression	3.88E-01
Estrogen Receptor Signaling	3.98E-01
HIF1α Signaling	5.72E-01
Wnt/Ca+ pathway	5.85E-01
DNA Double-Strand Break Repair by Non-Homologous End Joining	6.49E-01
Dendritic Cell Maturation	1.07E00
Endometrial Cancer Signaling	1.18E00
Wnt/β-catenin Signaling	1.51E00
DNA Double-Strand Break Repair by Homologous Recombination	1.59E00
VEGF Signaling	1.65E00
Small Cell Lung Cancer Signaling	2.05E00
Renal Cell Carcinoma Signaling	2.1E00
Regulation of the Epithelial-Mesenchymal Transition Pathway	2.24E00
Natural Killer Cell Signaling	2.47E00
PTEN Signaling	2.88E00
NF-κB Signaling	3E00
Ovarian Cancer Signaling	3.24E00
Molecular Mechanisms of Cancer	3.54E00
Noradrenaline and Adrenaline Degradation	3.61E00
Synaptic Long Term Depression	3.75E00
RAR Activation	3.82E00
Axonal Guidance Signaling	4.02E00
Gap Junction Signaling	4.04E00
IGF-1 Signaling	4.2E00
